# GCN5L1 regulates pulmonary surfactant production by modulating lamellar body biogenesis and trafficking in mouse alveolar epithelial cells

**DOI:** 10.1186/s11658-023-00506-0

**Published:** 2023-11-07

**Authors:** Wenqin Xu, Xiaocui Ma, Qing Wang, Jingjing Ye, Nengqian Wang, Zhenzhen Ye, Tianbing Chen

**Affiliations:** 1 Central Laboratory, Yijishan Hospital of Wannan Medical College, Wuhu, China; 2 Anhui Province Key Laboratory of Non-Coding RNA Basic and Clinical Transformation, Wannan Medical College, Wuhu, China; 3 Clinical Research Center for Critical Respiratory Medicine of Anhui Province, Wannan Medical College, Wuhu, China; 4 Henan Clinical Research Center of Childhood Diseases, Children’s Hospital Affiliated to Zhengzhou University, Zhengzhou, China; 5 Department of Pediatrics, Yijishan Hospital of Wannan Medical College, Wuhu, China

**Keywords:** Pulmonary surfactant, Type II alveolar epithelial cell, Lamellar body, Trafficking, GCN5L1

## Abstract

**Background:**

The pulmonary surfactant that lines the air–liquid surface within alveoli is a protein–lipid mixture essential for gas exchange. Surfactant lipids and proteins are synthesized and stored in the lamellar body (LB) before being secreted from alveolar type II (AT2) cells. The molecular and cellular mechanisms that regulate these processes are incompletely understood. We previously identified an essential role of general control of amino acid synthesis 5 like 1 (GCN5L1) and the biogenesis of lysosome-related organelle complex 1 subunit 1 (BLOS1) in surfactant system development in zebrafish. Here, we explored the role of GCN5L1 in pulmonary surfactant regulation.

**Method:**

GCN5L1 knockout cell lines were generated with the CRISPR/Cas9 system. Cell viability was analyzed by MTT assay. Released surfactant proteins were measured by ELISA. Released surfactant lipids were measured based on coupled enzymatic reactions. Gene overexpression was mediated through lentivirus. The RNA levels were detected through RNA-sequencing (RNA-seq) and quantitative reverse transcription (qRT)- polymerase chain reaction (PCR). The protein levels were detected through western blotting. The cellular localization was analyzed by immunofluorescence. Morphology of the lamellar body was analyzed through transmission electron microscopy (TEM), Lysotracker staining, and BODIPY phosphatidylcholine labeling.

**Results:**

Knocking out GCN5L1 in MLE-12 significantly decreased the release of surfactant proteins and lipids. We detected the downregulation of some surfactant-related genes and misregulation of the ROS–Erk–Foxo1–Cebpα axis in mutant cells. Modulating the activity of the axis or reconstructing the mitochondrial expression of GCN5L1 could partially restore the expression of these surfactant-related genes. We further showed that MLE-12 cells contained many LB-like organelles that were lipid enriched and positive for multiple LB markers. These organelles were smaller in size and accumulated in the absence of GCN5L1, indicating both biogenesis and trafficking defects. Accumulated endogenous surfactant protein (SP)-B or exogenously expressed SP-B/SP-C in adenosine triphosphate-binding cassette transporterA3 (ABCA3)-positive organelles was detected in mutant cells. GCN5L1 localized to the mitochondria and LBs. Reconstruction of mitochondrial GCN5L1 expression rescued the organelle morphology but failed to restore the trafficking defect and surfactant release, indicating specific roles associated with different subcellular localizations.

**Conclusions:**

In summary, our study identified GCN5L1 as a new regulator of pulmonary surfactant that plays a role in the biogenesis and positioning/trafficking of surfactant-containing LBs.

**Supplementary Information:**

The online version contains supplementary material available at 10.1186/s11658-023-00506-0.

## Introduction

The alveolar epithelium contains two distinct types of epithelial cells that support the gas exchange function of the respiratory system. Type I (AT I) cells, the main contributors to gas exchange, are flat and thin that cover ~ 95% of the alveolar surface [1]. Type II (AT II) cells are cuboidal and play an essential role in the synthesis and secretion of pulmonary surfactant (PS), a complex mixture consisting predominantly of phospholipids; ~ 10% of PS comprises specific surfactant proteins (SP-A, SP-B, SP-C, and SP-D) [2]. Surfactant lipids are synthesized in the endoplasmic reticulum (ER) and subsequently routed to the multivesicular body (MVB) and finally to the lamellar body (LB), the pulmonary surfactant storage organelle in AT II cells [2]. The transportation of surfactant lipids to LBs requires adenosine triphosphate (ATP)-binding cassette transporter A3 (ABCA3), a membrane-spanning transporter protein located on the limiting membrane of the LB [2]. Surfactant precursor proteins, including pro-SP-B and pro-SP-C, are processed proteolytically during trafficking to MVBs and LBs. Surfactant phospholipids are assembled with the active/mature hydrophobic proteins SP-B and SP-C into bilayer membranes that are stored in LBs [2, 3]. The contents of LBs are secreted into the airway via multiple stimulating factors. After secretion, the LB contents unwind and interact with SP-A and SP-D to produce tubular myelin and multilayered surface films that spread over the alveolus. The main function of this pulmonary surfactant system is to minimize the surface tension at the alveolar air–liquid interface, optimizing the mechanics of breathing and preventing alveolar collapse after expiration [2]. Mutations in several genes playing roles in regulating surfactant homeostasis have been associated with several severe lung diseases in neonates and older infants. The identification of surfactant proteins, lipid transporters, transcription factors, and signal pathways that regulate surfactant formation and metabolism has provided a framework for understanding the molecular and cellular processes of pulmonary surfactant production and homeostasis. Despite these advances, many aspects of the surfactant pathway remain unknown.

General control of amino acid synthesis 5 like 1 (GCN5L1), also known as biogenesis of lysosome-related organelles complex 1 subunit 1 (BLOS1), along with BLOS2 and Snapin, are shared subunits of two distinct octameric protein complexes—biogenesis of lysosome-related organelles complex 1 (BLOC-1) and BLOC-1-related complex (BORC) [4]. BORC regulates lysosomal vesicles positioning/trafficking [5]; BLOC-1 was identified earlier than BORC and participates in the biogenesis of lysosome-related organelles (LROs), including melanosomes in the retinal pigment epithelium and melanocytes, lytic granules in lymphocytes, delta granules in platelets, and lamellar bodies in alveolar type 2 epithelial cells [6]. Defects in BLOC-1 components are associated with Hermansky–Pudlak syndrome (HPS), which is a human autosomal recessive disorder characterized by oculocutaneous albinism and a deficiency of the platelet storage pool due to defective LRO biogenesis [6]. GCN5L1/BLOS1 has also been found to play a critical role in protein acetylation in the mitochondria and cytosol [7–12]; thus, this protein is involved in multiple aspects of cellular regulation. We previously generated and compared three zebrafish lines with mutations targeting two BLOC-1 and BORC shared subunits, GCN5L1/BLOS1 and BLOS2, and one BLOC-1 specific subunit, dystrobrevin binding protein 1a [13]. We confirmed the BLOC-1- and BORC-dependent roles of GCN5L1/BLOS1 in zebrafish larvae and identified the unique phenotype of absent swimbladder surfactant in GCN5L1 mutant fish [13]. Since the homology between the swimbladder and lung has long been recognized, here we explored the potential role of GCN5L1 in pulmonary surfactant regulation.

In this study, we confirmed that GCN5L1 is involved in surfactant regulation in murine alveolar epithelial cells. GCN5L1 knockout (KO) in MLE-12 cells impaired surfactant production and resulted in transcriptional changes in some genes associated with surfactant biology. The reactive oxygen species (ROS)–extracellular signal-regulated kinase (ERK)–forkhead box protein O1 (Foxo1)–CCAAT enhancer binding protein alpha (Cebpα) axis was found to be responsible for the dysregulation of some surfactant-related genes. Moreover, GCN5L1-KO MLE-12 cells contained small, misdistributed LB-like organelles. Hence, we revealed multiple ways that GCN5L1 regulates pulmonary surfactant production.

## Methods

### Generation of clonal GCN5L1 knockout cell lines with the CRISPR/Cas9 system

MLE-12 cells (American Type Culture Collection, CRL-2110) were cultured in DMEM/F12 medium supplemented with 10% (v/v) fetal bovine serum in a humidified 37 °C atmosphere containing 5% CO_2_. The px330–EGFP plasmid was derived from px330–mCherry (98750; Addgene), by replacing the mCherry with EGFP through double restriction enzyme digestion. Single-guide RNA sequences targeting mouse GCN5L1 were cloned into px330–EGFP. The plasmids were transiently transfected into cells using Lipofectamine 2000 reagent (Invitrogen). EGFP-positive cells were plated one cell per well in 96-well plates 48 h after transfection by flowcytometry-assisted cell sorting (FACS) (Beckman, MoFlo XDP), cultured, and analyzed by polymerase chain reaction (PCR) to confirm the homozygous KO of GCN5L1. The sgRNA and primer sequences are listed in Additional file 14: Table S3.

### MTT assay and measurement of released surfactant proteins and lipids

Cell viability was analyzed by 3-(4,5-dimethylthiazol-2-yl)-2,5-diphenyltetrazolium bromide (MTT) assay (Beyotime, Shanghai, China). Briefly, equal numbers of cells were seeded in 96-well plates and cultured under standard conditions. Before the assay, the cells were washed and further incubated with 0.5 mg/mL MTT for 4 h. The formed formazan dye was dissolved in dimethylsulfoxide (DMSO), and the absorbance at 570 nm was recorded.

Regarding the measurement of secreted surfactant proteins, cells were first counted and plated in 6-well plates, and fresh culture medium (DMEM/F12 with 2% FBS) was added when the cells reached 50% confluency. The cells were then cultured for another 2 days, and the supernatant was analyzed by enzyme-linked immunosorbent assay (ELISA) using kits for mouse SP-B and SP-C (CSB-E12638m and SB-E12639m, Cusabio, China).

For the quantification of released lipids, cells in 24-well plates at 90% confluence were washed with Ringer’s solution three times, 250 μL fresh solution containing 100 μM ATP was added, and the cells were cultured for 3 h. The supernatants were centrifuged and collected for the subsequent analysis of the phospholipid (the main component of surfactant lipids) content following a previous published method based on coupled enzymatic reactions [49]. Briefly, 100 μL of supernatant was first transferred into a 96-well plate well, 100 μL “reaction mix” containing choline oxidase (0.2 U/mL), horseradish peroxidase (HRP; 2 U/mL), phospholipase D (1 U/mL), and Amplex Red (0.1 mM) was added, and resorufin formation was recorded with a plate reader (Promega). The Amplex Red Phospholipase D Assay kit (A12219) was purchased from Thermo Scientific, and Phospholipase D (P0065) was purchased from Sigma.

### Lentivirus production and infection

The CDS sequences of mouse Gcn5l1, Foxo1, and Cebpα were amplified either from MLE-12 total cDNA (GCN5L1) or commercial plasmids (Origene MR227240 for Foxo1; Origene MR205482 for Cebpα), Mitochondria–GCN5L1 was generated by fusing the COX 8 mitochondrial targeting sequence (36 amino acid at N terminal) with the GCN5L1 encoding sequence and inserted into the pCDH-Puro lentiviral vector. The expression plasmids were cotransfected with packing vectors psPAX2 and pMD2.G into HEK293T cells using Lipofectamine 2000 (Invitrogen). The medium was collected ~ 50 h after transfection and filtered with a 0.45-µm filter. Viral infection was conducted with an enhancer (30001-2, Engreen). After infection, cells were selected with 2 µg/mL puromycin for 2 weeks. The primers used are listed in Additional file 14: Table S3.

### RNA sequencing and quantitative real-time PCR (qRT-PCR)

RNA sequencing and analyses were conducted by OE Biotech Co., Ltd. (Shanghai, China). Significantly differentially expressed genes (DEGs) between wild-type (WT) and GCN5L1-KO cells were identified based on fold change ≥ 2 and *P*-value ≤ 0.05 using the DEGseq function. Standard Gene Ontology (GO) and Kyoto Encyclopedia of Genes and Genomes (KEGG) analyses were performed. For qRT-PCR, total RNA was extracted using TRIzol reagent (Ambion, Life Technologies, CA, USA) according to the manufacturer’s protocol. RNA was reverse transcribed into cDNA using The RevertAid First Strand cDNA Synthesis Kit (Thermo Scientific, Lithuania, USA). qRT-PCR was performed according to the QuantiNova™ SYBR^®^ Green PCR Kit (Qiagen, Hilden, Germany) protocol. The primers are listed in Additional file 14: Table S3.

### Transmission electron microscopy (TEM), lysotracker staining, BODIPY phosphatidylcholine labeling, and measurement of ROS

For TEM analysis, cells were fixed in 2% glutaraldehyde, washed with cacodylate buffer, post-fixed in 1% OsO4, rewashed with cacodylate buffer, then placed in 1% uranyl acetate for 1 h and dehydrated in ethanol. The cell precipitate was then filtered through a propylene oxide/Epon series, and embedded in Epon. Hardened blocks were sectioned using an ultramicrotome (Leica), stained with uranyl acetate and lead citrate, and examined under a Hitachi HT-7700 electron microscope. The diameters of the LB-like organelles was measured using Image J.

For Lysotracker Red staining, the probes (Beyotime, Shanghai, China) were directly added to the cell medium and incubated for several minutes, then washed with fresh medium and imaged by confocal microscopy. For the BODIPY labeling assay, cells were incubated overnight with 1 μM β-BODIPY™ FL C_12_-HPC (D3792, Invitrogen) and washed before imaging.

For measuring intracellular ROS, cells were washed with phosphate-buffered saline (PBS) and incubated with 10 μM CM-H2DCFDA (Invitrogen) for 30 min. Cells were then trypsinized and resuspended in PBS containing 1% bovine serum albumin (BSA) and 0.5 mM ethylenediaminetetraacetic acid (EDTA).The analysis was conducted using an FC500 instrument (Beckman), and meanfluorescence intensity was normalized to the WT group. A series of concentrations of DPI and SCH772984 were tested for inhibiting ROS generation and ERK activity, respectively; 0.1 μM DPI (Sigma) and 0.5 μM SCH772984 (MCE) restored ROS and p-ERK levels in mutant cells to that in WT cells; thus, cells were incubated overnight with these concentrations before the subsequent analysis.

### Western blotting and immunofluorescence analyses

Cells were lysed in RIPA lysis buffer (Beyotime) containing complete protease inhibitor cocktail (Roche). Lysates were incubated on ice for 30 min and centrifuged to pellet the cellular debris. Samples were boiled for 10 min with SDS buffer, loaded onto 12% polyacrylamide gel, and transferred to PVDF membranes. Blots were incubated with primary antibodies (Actin, 1:5000 Cell Signaling Technology; Cebpα, Ttf1, Foxa2,Foxo1 and LC3 1:1000 Proteintech; p-ERK and total ERK, 1:1000 Cell Signaling Technology; SP-B, 1:1000 Abcam or 1:500 Santa cruz) followed by HRP-conjugated secondary antibodies.

For immunofluorescence microscopy, cells were seeded into confocal dishes and transiently transfected at 50% confluency with Sftpb, Sftpc, and TOM20 expressing plasmids (miaolingbio, P5954, P5862, and P24668, Wuhan, China). The GCN5L1–FLAG expressing fragment was PCR amplified by adding the flag sequence to the primer; ABCA3–EGFP and ABCA3–mCherry were constructed by amplifying three overlapping segments from the cDNA of human paracancerous lung tissue and cloning them into pEGFP-N1/pmCherry-N3 with an In-Fusion^®^ HD Cloning Plus kit from Takara (catalog no. 638909), cultured for another 36 h, fixed with 4% paraformaldehyde for 15 min, and permeabilized with 1% Triton X-100 for 10 min. For IF staining, fixed cells were incubated with primary antibodies (Lamp1, 1:200 Abcam; SP-B, 1:300 Abcam; SP-C, 1:300 Seven Hills Bioreagents; TOM20, 1:200 Proteintech; Flag, 1:200 CST) at 4 °C overnight. The next day, the cells were washed with PBS three times for 10 min, incubated for 1 h with the secondary antibody (Abcam), washed, and stained with DAPI before imaging.

## Results

### Knocking out GCN5L1 impaired surfactant production in MLE-12 cells

To investigate the potential function of GCN5L1 in surfactant biology, we examined its expression in the developmental lung using the Lung Gene Expression Analysis (LGEA) Web Portal database. We found that GCN5L1 is expressed in the mouse lung at all stages before and after birth (Additional file 1: Fig. S1A). Its expression was detected in different kinds of lung cells, including alveolar type II epithelial cells (Additional file 1: Fig. S1B). This finding was consistent with those of previous studies in which GCN5L1/BLOS1 and other components within the BLOC-1 complex were demonstrated to be widely expressed but with cell type-specific functions [14]. Next, we generated GCN5L1-mutant MLE-12 cells with the CRISPR/Cas9 system. Three targets for Cas9 were designed, and one with high efficiency, as evidenced by T7EI assay followed by sanger sequencing, was located near the start codon (T3, reverse strand) and used for subsequent experiments (Additional file 2: Fig. S2A-C). Multiple mutant clones were established (Additional file 2: Fig. S2D, Additional file 3: Fig. S3). M2 had a 43-bp deletion in one allele and a 146-bp deletion in the other; the M29 clone had 1-bp addition in one allele and a 4-bp deletion in the other (Additional file 2: Fig. S2D, Additional file 3: Fig. S3). We chose M2 and M29 for subsequent experiments, the mRNA levels were significantly decreased (Additional file 4: Fig. S4A), possibly through the nonsense-mediated decay mechanism. The successful KO of the GCN5L1 protein in these two mutant lines was also confirmed through western blotting (Additional file 4: Fig. S4B). Additional clones (M2, M8, M16, M20, M22, and M27) that contained nonframeshift mutations or WT sequences were discarded (Additional file 2: Fig. S2D, Additional file 3: Fig. S3).

**Fig. 1:**
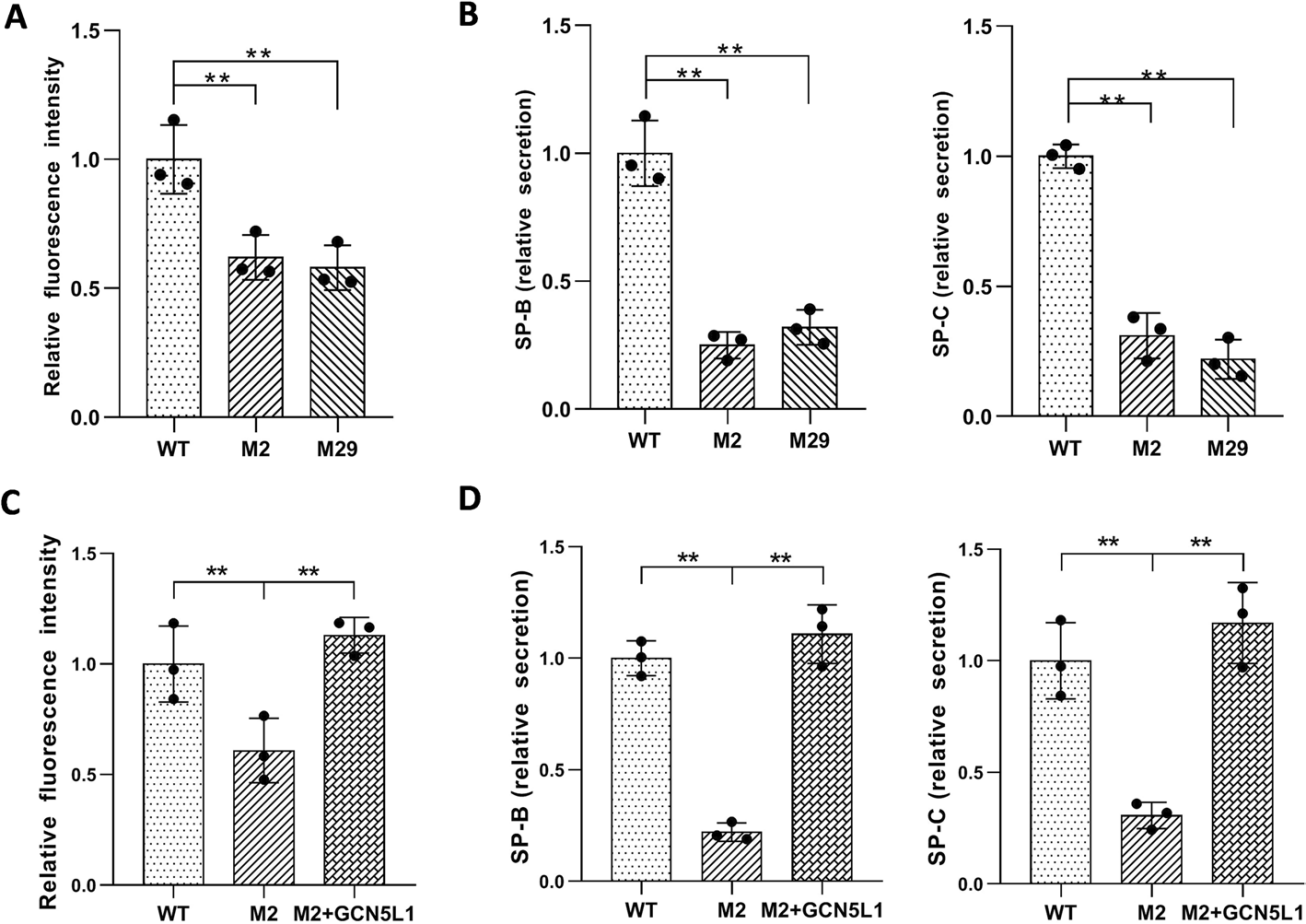
GCN5L1 gene KO impaired surfactant production in MLE-12 cells. **A** Phospholipid secretion in WT and GCN5L1-KO MLE-12 cells. **B** ELISAs of secreted SPs in the culture supernatant of WT and GCN5L1-KO MLE-12 cells. **C** Reconstruction of GCN5L1 expression repaired phospholipid secretion in GCN5L1 mutant cells. **D** Reconstruction of GCN5L1 expression repaired SP-B and SP-C secretion in GCN5L1 mutant cells. The results are expressed as the mean ± SD of three independent experiments; **P < 0.01; *t*-test

To assess the influence of GCN5L1 deletion on MLE-12 cells, we first tested cell viability using an MTT assay. The results showed no obvious effect on cell viability after GCN5L1 disruption (Additional file 4**:** Fig. S4C). Then, we analyzed the influence of GCN5L1 deficiency on surfactant production. The coupled enzymatic reactions method was used to quantify phospholipids, revealing significantly reduced phospholipid release from GCN5L1-mutant versus WT cells (Fig. 1A). Consistently, a comparative lipidomic analysis of the culture supernatant demonstrated a significant reduction in secreted phospholipid species from GCN5L1-mutant cells (Additional file 5**:** Fig. S5). The ELISA analysis also showed a significant reduction in released SP-B and SP-C in the culture supernatant from GCN5L1-mutant cells (Fig. 1B). Moreover, restoring GCN5L1 expression in M2 cells restored the level of released surfactant proteins and lipids (Fig. 1C, D). These results suggest that GCN5L1, similar to its function in regulating swimbladder surfactant in zebrafish, is involved in regulating lung surfactant production in mammalian cells.

### Disruption of GCN5L1 influenced the expression of genes associated with surfactant biology

As demonstrated in our previous work, GCN5L1/BLOS1 mutation resulted in the profound disruption of gene expression in the zebrafish swimbladder and hampered normal postembryonic development of the epithelium [13]. Hence, we performed RNA-seq to examine changes in the transcription profile after GCN5L1/BLOS1 disruption in MLE-12 cells. DEGs between control (WT) and M2 mutant cells were extracted (Additional file 6: Fig. S6A), and GO and KEGG enrichment analyses were conducted. Among the downregulated genes, the top enriched GO terms included “multicellular organism development,” “cell adhesion,” “cell differentiation,” and “growth factor binding” (Additional file 6: Fig. S6B). Regarding the upregulated genes, enriched GO terms included “toll-like receptor 4 signaling pathway,” and “positive regulation of transforming growth factor beta production” (Additional file 6: Fig. S6C). In the KEGG analysis, the DEGs were mostly enriched in “focal adhesion,” “ECM-receptor interaction,” and “TGF-beta signaling pathway” (Additional file 6: Fig. S6D-E). These enriched terms could function in maintaining epithelial characteristics and/or AT II cell specific functions.

**Fig. 2:**
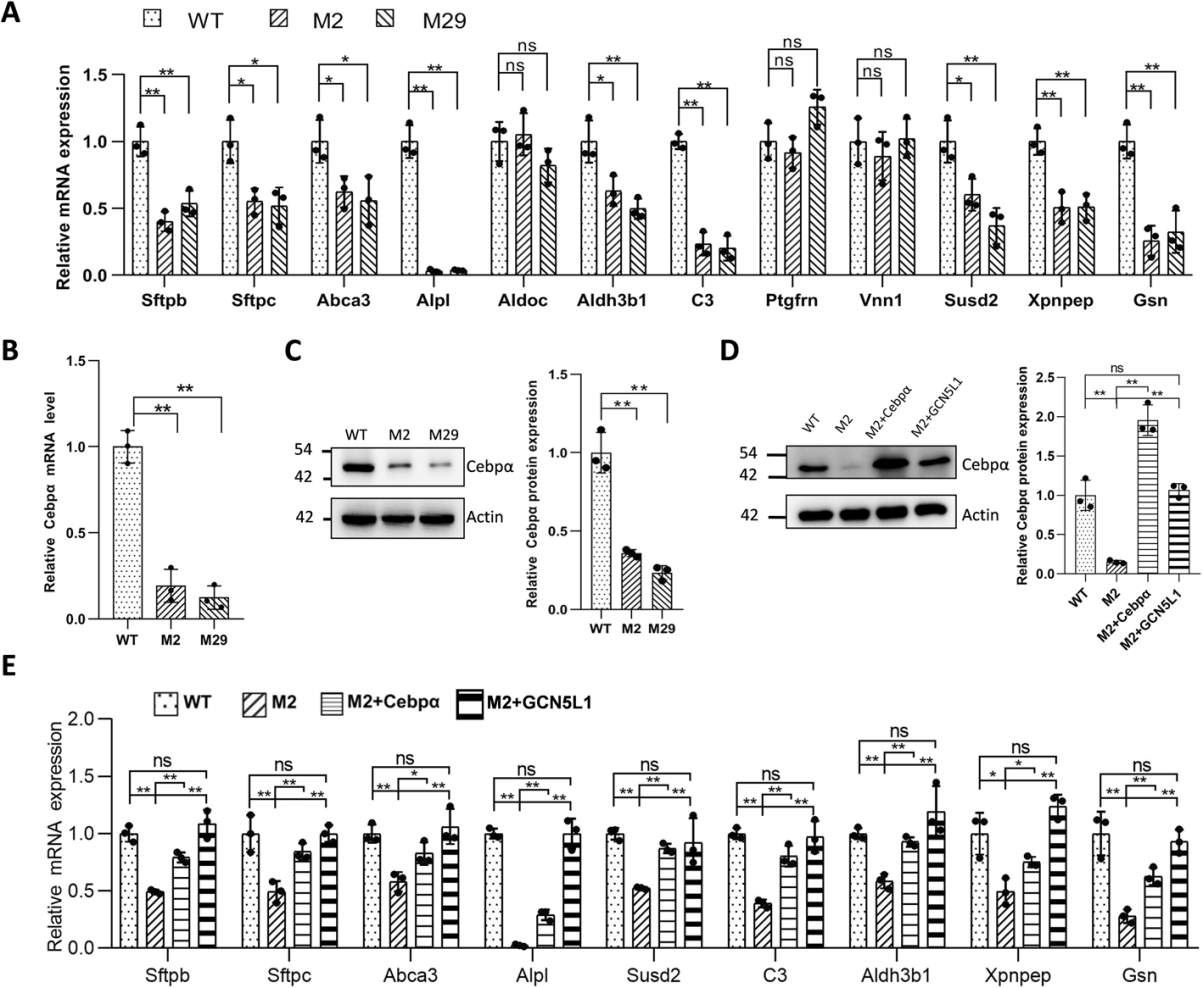
Disruption of GCN5L1 altered the expression of Cebpα and surfactant-related genes. **A** Q-PCR verification of the surfactant-related DEGs in GCN5L1 mutant clones. **B** Q-PCR detection of Cebpα mRNA expression in WT and GCN5L1-KO MLE-12 cells. **C** Representative immunoblot image of Cebpα protein expression levels in WT and GCN5L1-KO MLE-12 cells. **D** Representative immunoblot images of Cebpα protein levels in WT cells, M2 cells, M2 cells infected with lentivirus expressing exogenous Cebpα (M2 + Cebpα), and M2 cells infected with lentivirus expressing exogenous GCN5L1 (M2 + GCN5L1). **E** Q-PCR assays of the RNA levels of surfactant-related genes in WT cells, M2 cells, and M2 cells infected with lentivirus expressing exogenous Cebpα (M2 + Cebpα), and M2 cells infected with lentivirus expressing exogenous GCN5L1 (M2 + GCN5L1). The results are expressed as the mean ± SD of three independent experiments; ns, not significant;*P < 0.05, **P < 0.01; *t*-test

To further investigate the surfactant defect in the mutant cells, we mined DEGs related to surfactant biology. The expression levels of surfactant protein genes *Sftpb* and *Sftpc* and the lipid transporter *Abca3* were reduced in M2 cells (Additional file 12: Table S1). However, no *Sftpa1* or *Sftpd* expression was detected in WT or mutant cells (Additional file 12: Table S1). Since SP-B, SP-C, and Abca3 are LB components, we further examined the expression of other LB proteins by merging the LB proteome with our DEGs [15, 16], revealing more downregulated LB genes, including *Alpl*, *Aldoc*, *Aldh3b1*, *C3*, *Ptgfrn*, *Vnn1*, *Susd2*, *Xpnpep*, and *Gsn* (Additional file 12: Table S1). Q-PCR confirmed the dysregulation of *Sftpb*, *Sftpc* and *Abca3*, and some other LB genes in M2 and M29 cells (Fig. 2A). Additionally, we examined the mRNA expression of some transcription factors involved in surfactant production [17–21]. No significant changes in the expression levels of *Nkx2-1*, *Foxa2*, *Gata6*, *Nfatc3*, *Stat3*, *Srebf1*, and *Srebf2* were observed after GCN5L1 deletion (Additional file 13: Table S2). However, the expression level of Cebpα was significantly decreased, as demonstrated by RNA-seq (Additional file 13: Table S2) and confirmed by q-PCR and western blotting (Fig. 2B, C). This decline in Cebpα expression was restored in lentivirus-mediated GCN5L1-reconstructed M2 cells (Fig. 2D). Moreover, lentivirus mediated overexpression of both Cebpα and GCN5L1 significantly restored the downregulated mRNA levels of *Sftpb*, *Sftpc* and other LB-associated genes in GCN5L1 mutant cells (Fig. 2D, E). In summary, these results suggest that GCN5L1 deletion dysregulates genes involved in surfactant biology, and Cebpα is a crucial associated regulator.

### Disruption of GCN5L1 altered the activity of the ROS-ERK-Foxo1-Cebpα axis in MLE-12 cells

**Fig. 3:**
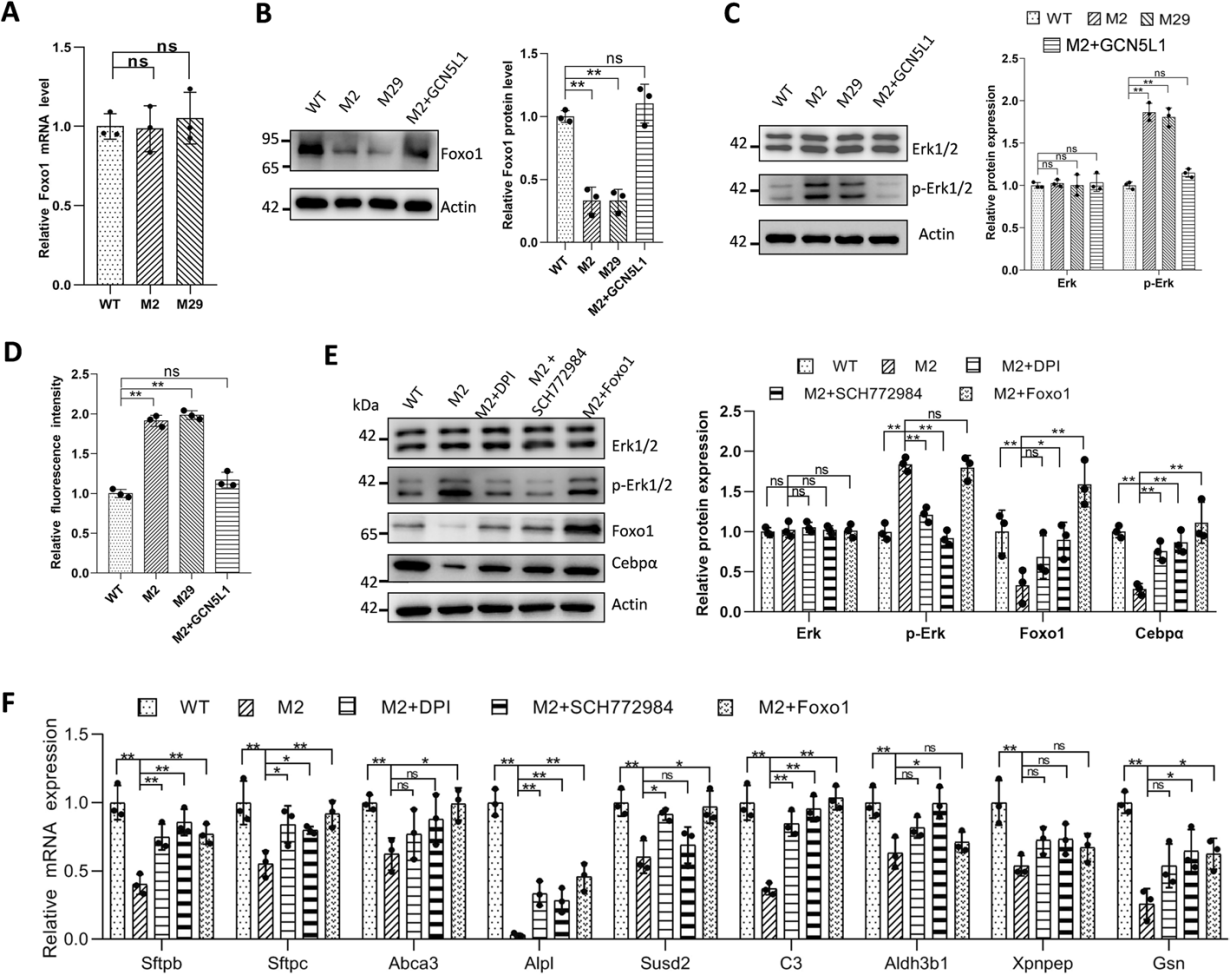
Disruption of GCN5L1 altered the activity of the ROS–ERK–FOXO1 axis in MLE-12 cells. **A** Q-PCR detection of Foxo1 mRNA expression in WT, GCN5L1-KO MLE-12 cells. **B** Representative immunoblot image of Foxo1 protein expression levels in WT, GCN5L1-KO MLE-12 cells and M2 cells infected with lentivirus expressing exogenous GCN5L1 (M2 + GCN5L1). **C** Representative immunoblot images of ERK and p-ERK protein expression levels in WT, GCN5L1-KO MLE-12 cells and M2 cells infected with lentivirus expressing exogenous GCN5L1 (M2 + GCN5L1). **D** Cellular ROS levels of WT, GCN5L1-KO MLE-12 cells, and M2 cells infected with lentivirus expressing exogenous GCN5L1 (M2 + GCN5L1). Cells were incubated with CM-H2DCFDA and measured by FACS; mean fluorescence intensity was normalized to WT group. **E** Representative immunoblot images of ERK, p-ERK, Foxo1, and Cebpα protein expression levels in WT cells, M2 cells treated with DPI (M2 + DPI) or SCH772984 (M2 + SCH772984), and M2 cells infected with lentivirus expressing exogenous Foxo1 (M2 + Foxo1). **F** Q-PCR assays of the RNA levels of surfactant-related genes in WT cells, M2 cells treated with DPI (M2 + DPI) or SCH772984 (M2 + SCH772984), and M2 cells infected with lentivirus expressing exogenous Foxo1 (M2 + Foxo1). The results are expressed as the mean ± SD of three independent experiments; ns, not significant; *P < 0.05,**P < 0.01; *t*-test

To explore the upstream mechanism underlying Cebpα dysregulation, we first examined the protein levels of Nkx2-1 and Foxa2, both of which are known Cebpα regulators [21]. Consistent with the mRNA findings (Additional file 13: Table S2), no significant changes in the expression levels of these two proteins were detected in GCN5L1 mutant cells (Additional file 7: Fig. S7A). We next used the STRING database to search for potential Cebpα interactors. Ten primary interactors were found (Additional file 7: Fig. S7B), among which Foxo1 attracted our attention since a previous study revealed that the ROS-ERK-Foxo1 axis is dysregulated in GCN5L1-deficient hepatocytes [22]. Moreover, Foxo1 could interact with Cebpα and influence its expression [23, 24]. Thus, we examined the activity of this axis. While *Foxo1* mRNA expression levels were similar (Fig. 3A), the protein levels in mutant cells were significantly reduced (Fig. 3B). ERK activity was also analyzed; consistently, ERK was found to be activated, as indicated by increased p-ERK levels (Fig. 3C). ROS level elevation after GCN5L1 deletion has been reported in different circumstances [7, 25–27]. As expected, the ROS levels were also increased in mutant MLE-12 clones (Fig. 3D). Moreover, the activity of the ROS–ERK–Foxo1 axis recovered in GCN5L1-reconstructed M2 cells (Fig. 3B–D). Together, these data indicate that alteration of ROS–ERK–Foxo1–Cebpα axis activity occurs following GCN5L1 depletion in MLE-12 cells.

To assess whether alteration of the ROS–ERK–Foxo1–Cebpα axis was responsible for the surfactant defect in GCN5L1 mutant cells, we assessed the effect of modifying different components within the axis. As shown in Fig. 3E, reducing ROS with diphenylene iodonium (DPI) suppressed ERK activation and increased Foxo1 expression in M2 cells. Inhibiting ERK activity with SCH772984 also restored Foxo1 levels. These two modifications together with lentivirus mediated overexpression of Foxo1 all significantly promoted Cebpα protein expression and partially restored the expression of most dysregulated surfactant related genes (Fig. 3E, F). However, unexpectedly, the effect of these rescue experiments and Cebpα overexpression did not significantly affect surfactant production (Additional file 8: Fig. S8A-D). Collectively, these results imply that the ROS–ERK–Foxo1–Cebpα axis is involved in the misregulation of some surfactant related genes in GCN5L1-mutant cells.

**Fig. 4:**
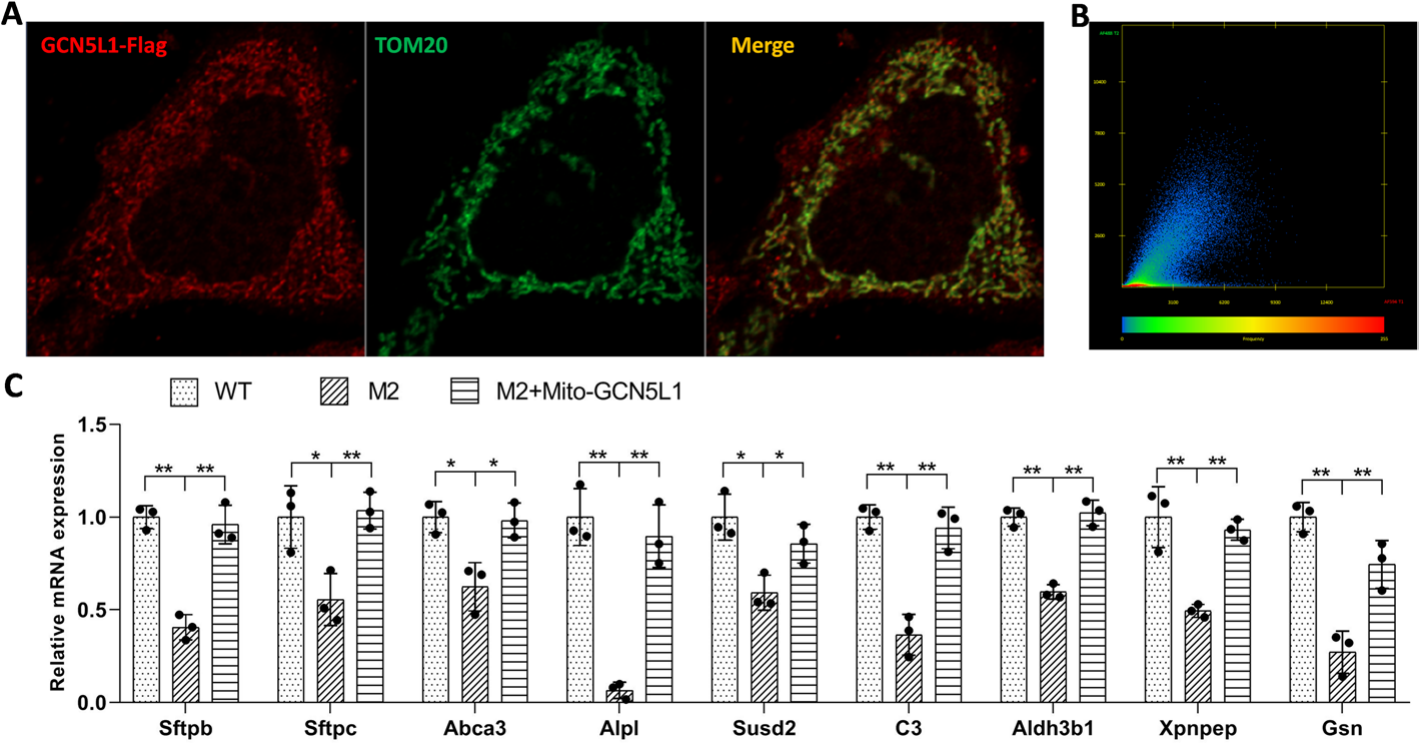
Mitochondria-localized GCN5L1 is responsible for the regulation of surfactant-related genes. **A** Colocalization of GCN5L1–FLAG with TOM20 in MLE-12 cells. Cells were cotransfected with TOM20- and GCN5L1–FLAG-expressing plasmids before staining with anti-TOM20 and anti-FLAG antibodies. **B** The colocalization scatter plot of anti-TOM20 and anti-GCN5L1–FLAG signals. Pearson correlation coefficient is 0.78, Manders’ overlap coefficient is 0.82. **C** Q-PCR assays of the RNA levels of surfactant related genes in WT cells, M2 cells, and M2 cells infected with lentivirus expressing mito-GCN5L1 (M2 + mito-GCN5L1).The results are expressed as the mean ± SD of three independent experiments; *P < 0.05, **P < 0.01; *t*-test

Given that GCN5L1 is repeatedly reported to be a mitochondrial enriched protein [7–12], we tested its localization in MLE-12 cells. Consistent with previous reports, we observed strong colocalization of GCN5L1–FLAG with the mitochondrial marker TOM20 in MLE-12 cells (Fig. 4A, B). Thus, we tried to restore the GCN5L1-KO defect through the direct reconstruction of GCN5L1 expression in mitochondria. Unexpectedly, unlike GCN5L1 reconstruction (Fig. 1C, D), mito-GCN5L1 reconstruction failed to significantly restore surfactant release (Additional file 8: Fig. S8E–F); however, it rescued the expression level of dysregulated genes in mutant cells (Fig. 4C), indicating a specific role of mitochondria-localized GCN5L1 in the regulation of these genes.

### Disruption of GCN5L1 impaired lamellar body biogenesis and trafficking in MLE-12 cells

**Fig. 5:**
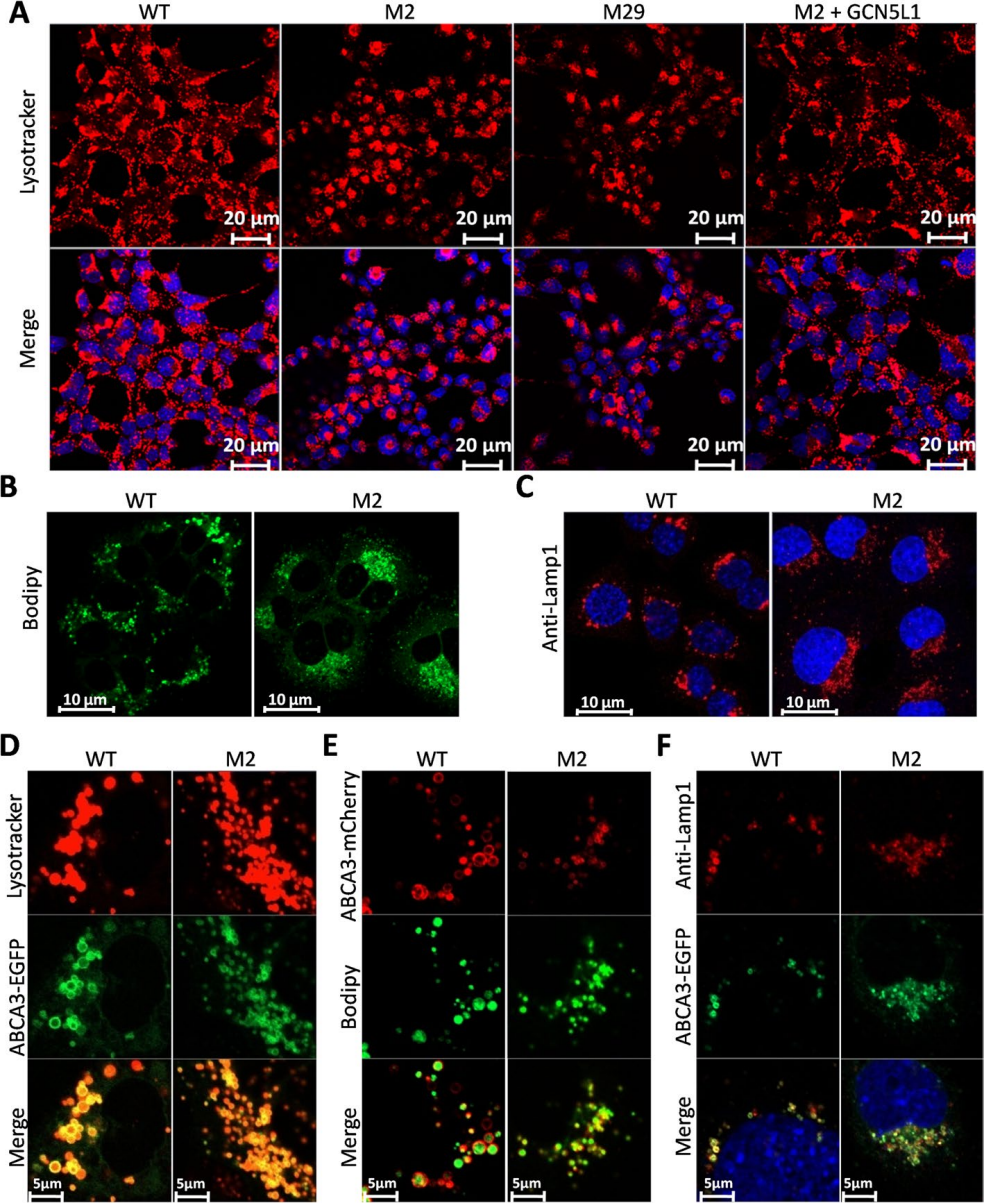
Impairment of LB positioning/trafficking in GCN5L1-KO cells. **A** Lysotracker Red staining of WT, GCN5L1-KO clones (M2 and M29), and GCN5L1 reconstructed mutant cells (M2 + GCN5L1). **B** WT and M2 cells were metabolically labeled with BODIPY phosphatidylcholine. **C** WT and M2 cells were immunostained with Lamp1 antibody. **D–F** WT and M2 cells were transfected with h-ABCA3–EGFP or ABCA3–mCherry before staining with Lysotracker Red (**D**), BODIPY phosphatidylcholine (**E**), and Lamp1 antibody (**F**)

The above findings do not explain the decreased release of surfactant from mutant cells. Surfactant lipids and proteins are transported and stored in LBs before secretion. Moreover, a previous study demonstrated that GCN5L1/BLOS1 is a subunit of the BORC protein complex that plays a role in lysosomal organelle positioning/trafficking [5, 28–30]. Since the LB is an LRO and shares some common features with lysosomes [31], we explored potential LB positioning/trafficking defects after GCN5L1 mutation. Lysotracker Red was used to stain LB-like organelles in MLE-12 cells [32–34]. Strikingly, while these lysotracker-positive organelles were scattered in WT MLE-12 cells, they were perinuclearly clustered in GCN5L1 mutant M2 and M29 cells (Fig. 5A, Additional file 9: Fig. S9A). This phenotype was rescued in M2 cells when the expression of GCN5L1 was reconstructed (Fig. 5A, Additional file 9: Fig. S9A). The colocalization of these signals within ABCA3–EGFP vesicles indicated they were likely LBs (Fig. 5D, Additional file 9: Fig. S9D). To further confirm the identity of these organelles, we performed metabolic labeling with BODIPY phosphatidylcholine (a probe that undergoes native-like transport and metabolism in cells). As shown in Fig. 5B, these organelles were also lipid-enriched, similar to LBs in primary AT2 cells. BODIPY-labeled vesicles were also ABCA3–mCherry positive and showed accumulation in mutant cells (Fig. 5B, E, Additional file 9: Fig. S9B, E). Endogenous Lamp1, a frequently reported marker for LBs, was found to colocalize well with ABCA3–EGFP (Fig. 5C, F). Similarly, these Lamp1-positive structures also accumulated in GCN5L1 mutant cells (Fig. 5C, F, Additional file 9: Fig. S9C, F). Because autophagosomes are also stained by Lysotracker [35], and previous studies have revealed abnormal autophagosome accumulation in GCN5L1 deleted cells [36, 37], we investigated whether the Lysotracker Red stained organelles were or contained autophagosomes. Transient transfection of a GFP–LC3-expressing plasmid failed to label these organelles in GCN5L1 mutant cells (Additional file 10: Fig. S10A), suggesting they were not autophagosomes. Immunoblotting with LC3 antibody also showed no obvious alteration in the LC3 II/I ratio after GCN5L1 knockout (Additional file 10: Fig. S10B). Collectively, these results indicate that LB-like vesicles accumulate in MLE-12 cells after GCN5L1 disruption.

**Fig. 6:**
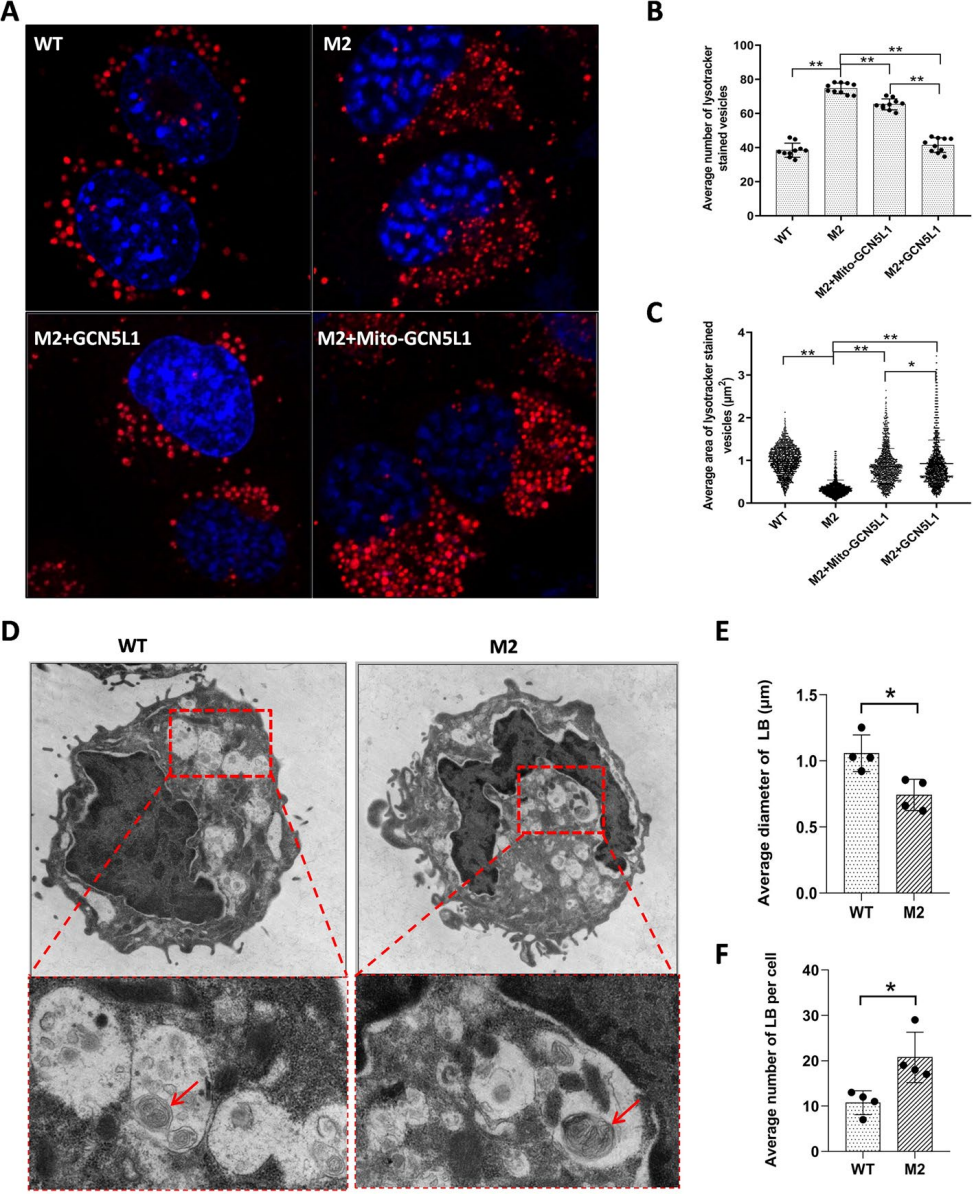
Abnormal size and number of LB-like organelles in GCN5L1-KO cells. **A** Representative images showing Lysotracker-stained organelles in WT, M2, M2 + GCN5L1, and M2 + mito-GCN5L1 cells. **B**, **C** The numbers and size (area) in WT, M2, M2 + GCN5L1, and M2 + mito-GCN5L1 cells; **P < 0.01 (Kolmogorov–Smirnov test). **D** Transmission electron micrographs of WT and M2 cells. Many LB-like organelles are present within the cytoplasm; arrows point to lamellar-like materials in the organelles. **E**, **F** The diameters and numbers of LBs in the TEM images were measured and counted with Image J. The results are expressed as the mean ± SD of four independently measured data; *P < 0.05; t-test

**Fig. 7:**
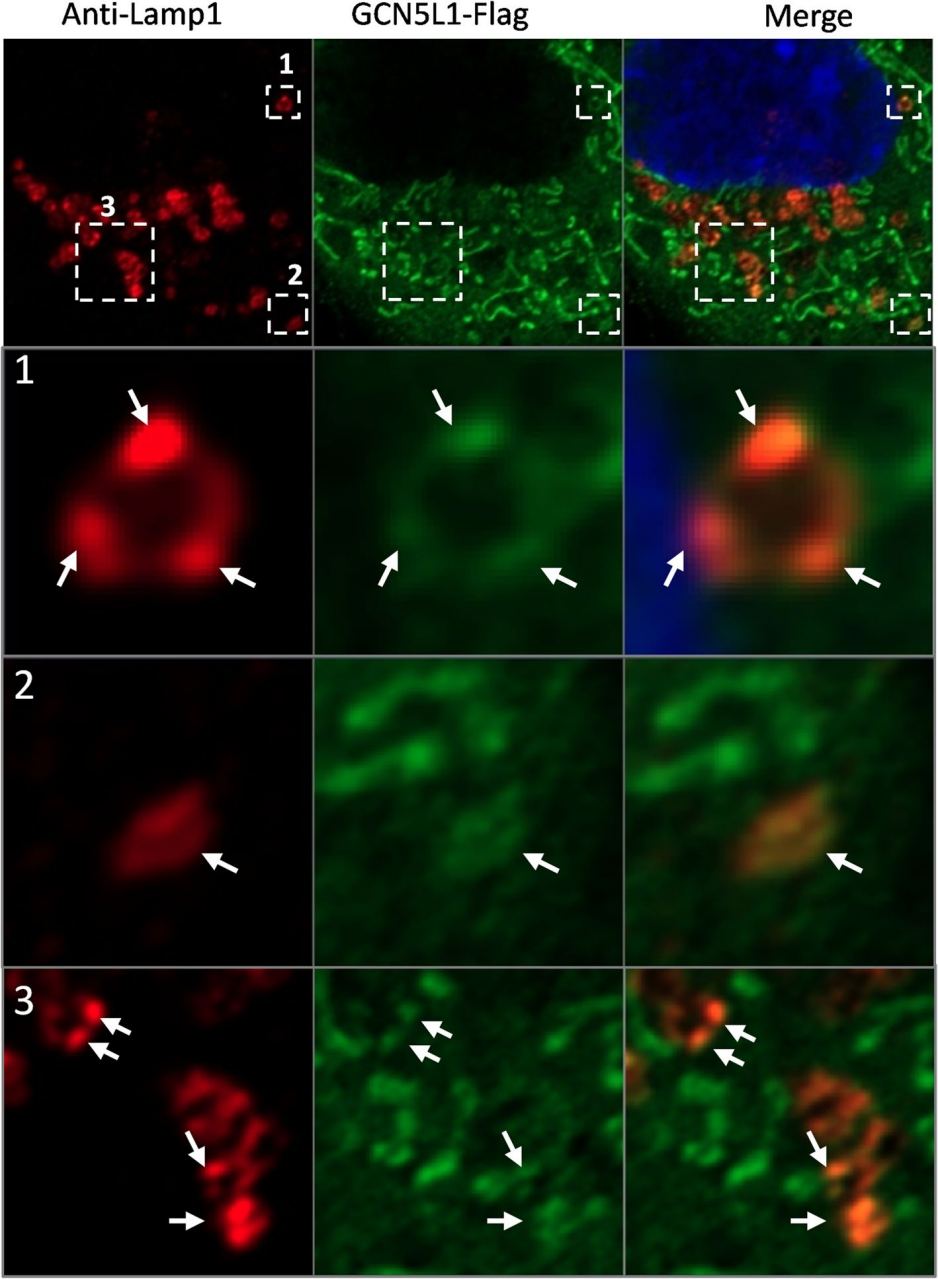
Localization of GCN5L1 in LBs. Cells were transfected with GCN5L1–FLAG-expressing plasmid and stained with anti-Lamp1 and anti-FLAG antibodies

The size and number of these organelles differed between WT and mutant cells. Careful quantification revealed found that while the number of these LBs increased in mutant cells, their size significantly decreased (Fig. 6A–C). This phenotype indicated an LB biogenesis defect and is consistent with the downregulation of multiple LB-related genes in mutant cells (Fig. 2A). Interestingly, we found that despite the lack of increased surfactant release (Additional file 8: Fig. S8E–F), the mito-GCN5L1 expressing M2 cells had normal-sized LBs, indicating a direct role of mito-GCN5L1 in LB biogenesis (Fig. 6A, C). These restored LBs in mito-GCN5L1-expressing cells failed to efficiently transport at the same level as those in WT or GCN5L1 reconstructed M2 cells, resulting in accumulation within cells (Fig. 6A, B). Considering that no significant increase in surfactant release was observed, the relatively smaller number of LBs in mito-GCN5L1-expressing cells than that in M2 cells could have resulted from incomplete counting since accumulated large LBs partially overlapped in the images. Next, we performed TEM analysis on WT and M2 cells to better examine these organelles. As shown in Fig. 6D and E, MLE-12 M2 cells contained more but smaller LB-like organelles containing numerous membranous structures potentially constituting cell surfactant. However due to the relatively low activity of the surfactant biogenesis machinery in these cells, these membranous structures did not fill the LBs. Collectively, these results indicate that the disruption of GCN5L1 impaired LB biogenesis and positioning in MLE-12 cells. Additionally, the GCN5L1 signal clearly colocalized with the endogenous LB marker Lamp1 (Fig. 7, Additional file 11: Fig. S11), indicating a possible direct role of GCN5L1 in regulating the trafficking of these organelles.

### Disruption of GCN5L1 resulted in the accumulation of surfactant proteins within MLE-12 cells

**Fig. 8:**
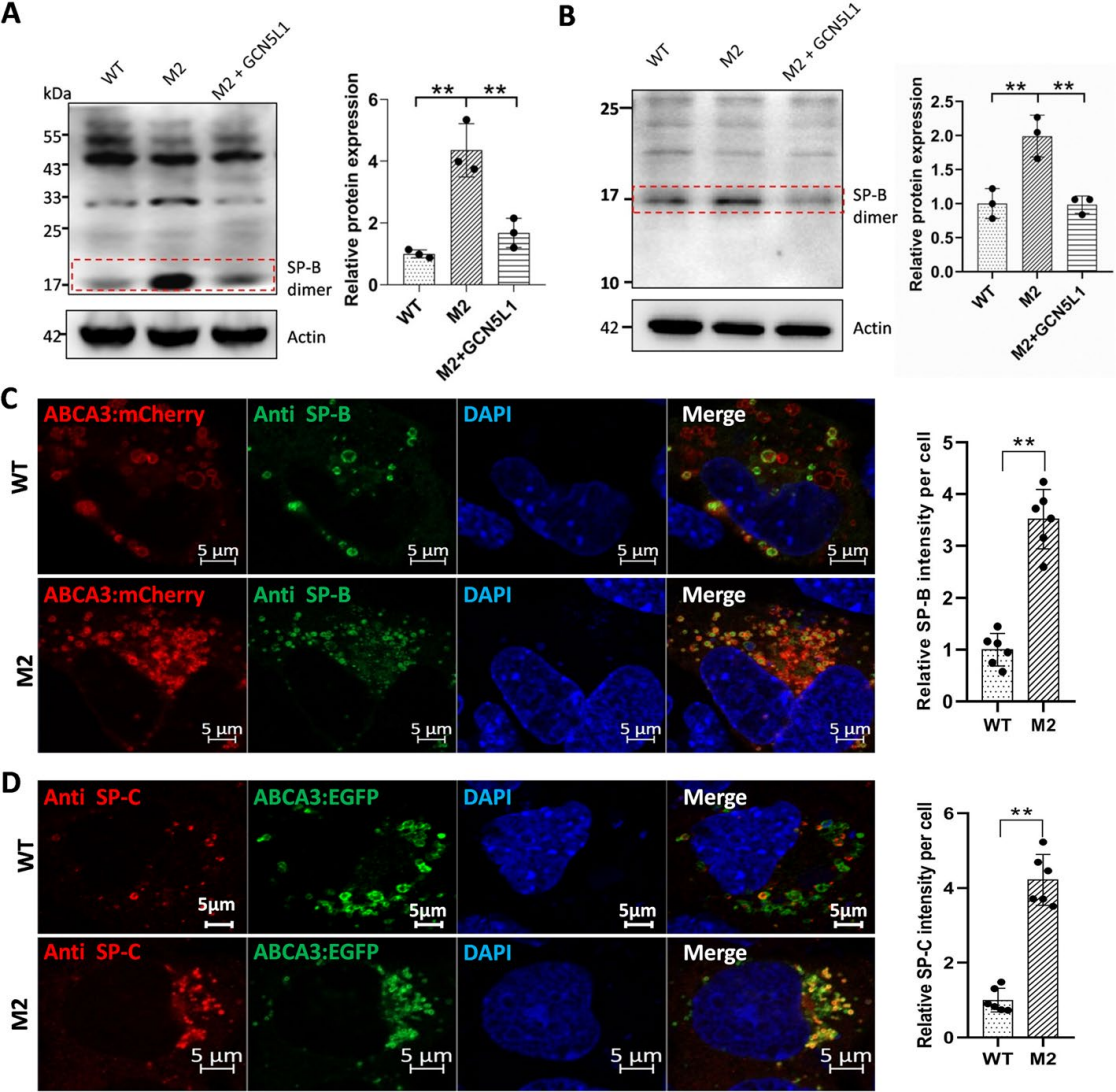
Accumulation of SP proteins in GCN5L1-KO cells. **A** Immunoblot image of WT, M2, and GCN5L1 reconstructed M2 + GCN5L1 cells with an SP-B antibody (Santa Cruz). **B** Immunoblot image of WT, M2, and GCN5L1 reconstructed M2 + GCN5L1 cells with another SP-B antibody (Abcam). **C** WT and M2 cells were cotransfected with h-ABCA3–mCherry and m-Sftpb expressing plasmids and immunostained with an SP-B antibody (Abcam). **D** WT and M2 cells were cotransfected with h-ABCA3–EGFP- and m-Sftpc-expressing plasmids and immunostained with an SP-C antibody (Seven Hills Bioreagents). **P < 0.01; *t*-test

To investigate the consequences of LB defects on surfactant production, especially surfactant protein secretion, we performed western blotting to examine the endogenous protein levels of SP-B and SP-C. Although mature SP-C was not detected in either control or mutant cells (data not shown), we detected a striking ~ 18KD band, corresponding to the SP-B dimer [38–40], with two different SP-B antibodies (Fig. 8A, B). While the expression of this band was weak in WT and GCN5L1-reconstructed M2 cells, it was strong in GCN5L1-mutant cells, implying accumulation of this protein within mutant cells. We further visualized the accumulated proteins within cells directly through immunofluorescence. Neither endogenous SP-B nor SP-C could be detected with our antibodies. However, when the cells were pretransfected with SP-B- or SP-C-expressing plasmids, we detected more perinuclear signals located within ABCA3-positive organelles in mutant versus control cells (Fig. 8C, D). Collectively, these results indicate that defects resulting from GCN5L1 deletion caused the accumulation of surfactant proteins within LBs in MLE-12 cells.

## Discussion

**Fig. 9:**
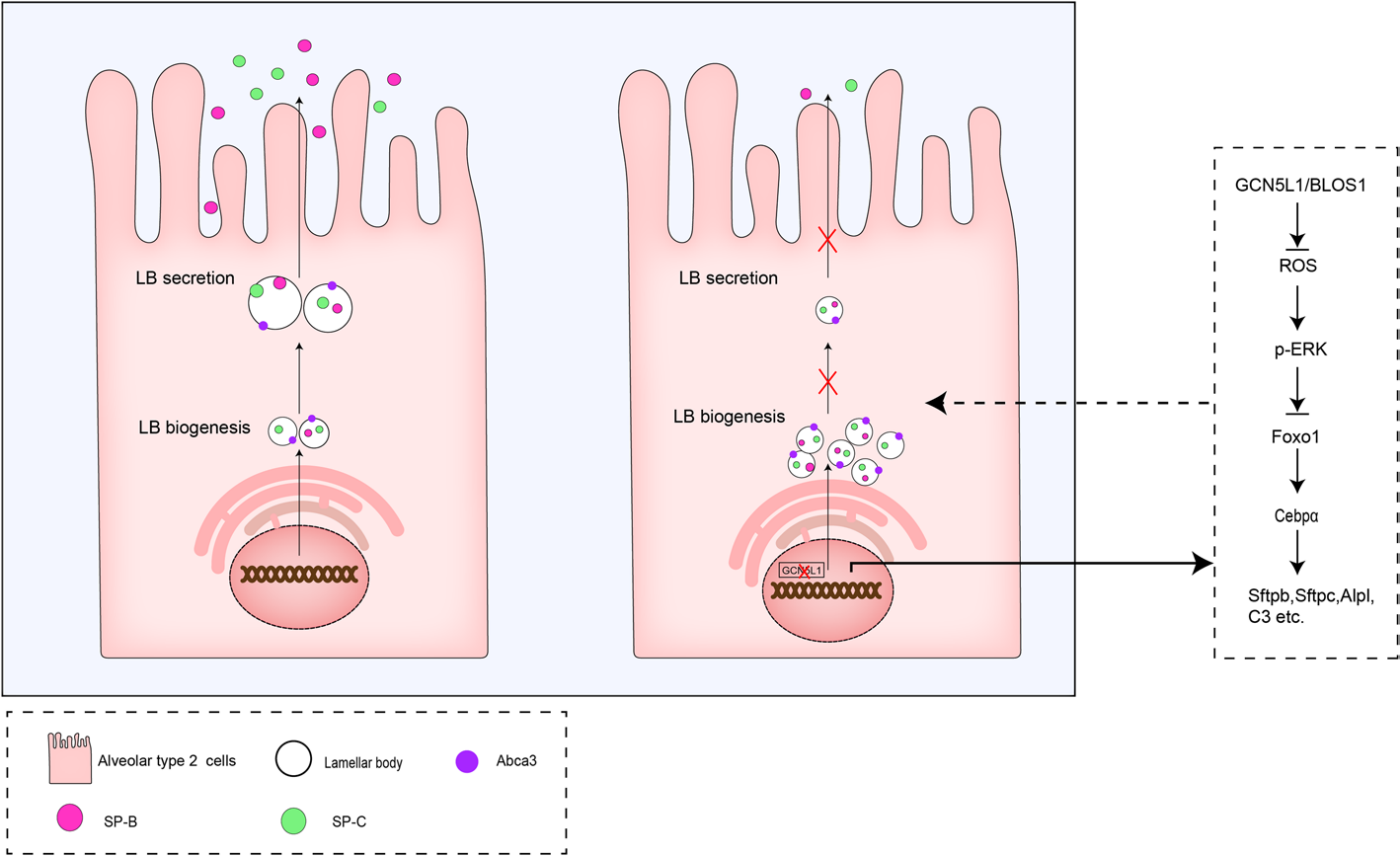
A hypothetical illustration of GCN5L1 functions in the regulation of pulmonary surfactant production

In the present study, we investigated the roles of GCN5L1 in pulmonary surfactant regulation by establishing GCN5L1-KO MLE-12 (murine AT II-like) cell lines. The release of surfactant proteins and lipids from GCN5L1-KO cells was significantly decreased. An enrichment analysis of RNA-seq data revealed the alteration of pathways likely influencing epithelial functions. We revealed the “ROS–Erk–Foxo1–Cebpα” axis downstream of GCN5L1, which regulates the expression of some LB-related genes and contributes to the morphology defects in mutant cells during LB biogenesis (Fig. 9). Moreover, we identified that LBs in mutant cells were defective in trafficking, thus accumulating surfactant proteins within GCN5L1-KO cells (Fig. 9).

Defective surfactant production was observed in two independent GCN5L1-KO clones, and the defect could be rescued when GCN5L1 expression was reconstructed (Fig. 1). Hence, the genotype-to-phenotype relationship was confirmed. Our previous work in zebrafish showed disturbed surfactant-related gene expression in the GCN5L1/BLOS1-KO swimbladder [13]. We obtained similar results in this study. RNA-seq identified several downregulated genes related to surfactant biology, including some LB-associated genes, such as *Sftpb* and *Sftpc*, and the transcription factor Cebpα (Fig. 2). The decreased supply of these LB components, and possibly other yet unknown factors involved in LB biogenesis, explains the smaller LBs in GCN5L1-KO cells (Figs. 6, 9). The trafficking defect could explain the relatively higher number of LBs in GCN5L1-KO cells (Fig. 6). Similarly, the immature LBs accumulated in the swimbladder epithelium of GCN5L1/BLOS1-KO zebrafish [13]. The regulatory effect of Cebpα on surfactant-related genes has been previously reported [21]. In this study, we also detected synchronous changes in *Sftpb*, *Sftpc* and *Abca3* levels as Cebpα levels changed (Fig. 2A, E). Moreover, the mRNA levels of several unreported LB genes fluctuated following changes in Cebpα expression in mutant cells (Fig. 2A, E). Hence, these genes could also be downstream targets of Cebpα. GO and KEGG analyses also revealed enriched pathways that could influence epithelial and/or AT II cell function (Additional file 6**:** Fig. S6). We speculate that GCN5L1 plays a vital role in fetal lung development. Our previous work showed normal early embryonic development but defective postembryonic swimbladder development in GCN5L1/BLOS1-KO zebrafish [13]. Thus, the potential role of GCN5L1 in late stage of lung development (such as the alveolarization stage) should be investigated in the future.

Changes in the activity of the “ROS–Erk–Foxo1” axis was previously reported in GCN5L1-KO hepatocytes [22]. Dysregulation of this axis also occurred in GCN5L1-KO MLE-12 cells (Fig. 3), implying that this pathway and the regulatory effect of GCN5L1 on it is similar in these two cell types. Indeed, increased ROS after GCN5L1 KO has been previously reported in several studies [7, 25–27]. Mitochondria are a main source of ROS and GCN5L1 has been repeatedly reported as a mitochondria enriched protein [4]; thus, we hypothesized that this localization would be associated with ROS control. Consistently, we found mitochondrial enrichment of GCN5L1 in MLE-12 cells (Fig. 4A). Autophagy changes have been documented in GCN5L1-KO MEF cells [36, 37]. Because autophagy also affects LB biogenesis [41], we analyzed the autophagy activity. No obvious alteration of autophagesome formation was observed after GCN5L1 KO (Additional file 10**:** Fig.S10), suggesting that the ability of GCN5L1 to regulate autophagy is cell type-dependent and that the autophagy pathway is not involved in the surfactant defect in GCN5L1-KO MLE-12 cells.

As mentioned in the introduction section, GCN5L1 is a subunit of the BLOC-1 complex. This complex (comprising eight distinct proteins: BLOS1/GCN5L1, BLOS2, BLOS3, Snapin, Dysbindin, Pallidin, Muted, and Cappuccino) was identified as part of the vesicular transport machinery 2 decades ago [42]. Despite the ubiquitous expression of its components in multiple organs, the main known function of the complex has long been restrained to the biogenesis of cell type-specific LROs [31]. This was due to the well-studied connection between BLOC-1 mutations and the human disease HPS, an autosomal recessive disorder characterized primarily by oculocutaneous albinism and a deficiency in the platelet storage pool resulting from defective LRO biogenesis, including melanosomes in the retinal pigment epithelium or melanocytes, and delta granules in platelets [6]. Loss-of-function mutations in BLOS3, Dysbindin, Pallidin, Muted, and Cappuccino correspond to different HPS subtypes in humans or cause HPS-like phenotypes in mice [31]. LBs in alveolar type 2 epithelial cells are LROs, and defects in these organelles have been proposed in some subtypes of HPS [31]; however, to the best of our knowledge, no studies have reported BLOC-1 deficiency with an LB/surfactant phenotype in mice or HPS patients. Thus, the roles of GCN5L1 in lung LB/surfactant regulation identified here are likely BLOC-1 independent. Our previous work in zebrafish also supports the BLOC-1-independent role of this protein in regulating swimbladder surfactant [13].

Moreover, unlike the five HPS proteins, dysfunction of the remaining three proteins of BLOC-1 (BLOS1, BLOS2, or Snapin) is lethal in mice, indicating novel functions beyond BLOC-1 [37, 43, 44]. Interestingly, BLOS1, BLOS2, and Snapin (also named BORCS1-3) comprise a subunit of another complex, BORC, which contains an additional five subunits (also named BORCS4-8), namely KXD1, myrlysin (LOH12CR1), lyspersin (C17orf59), diaskedin (C10orf32), and MEF2BNB [5]. BORC promotes “lysosome” (late endosomes and lysosomes) movement toward the cell periphery, and, thereby, affects lysosome positioning in HeLa cells [5]. Considering that the LB is a lysosome-related organelle and shares some properties with lysosomes, we examined potential transport defects in GCN5L1-KO MLE-12 cells. Although not shown in previous reports [38], we observed LB-like organelles in MLE-12 cells (Figs. 5, 6). These organelles were both positive for Abca3 (exogenous) and Lysotracker Red, similar to previously documented LBs in primary human type II cells [34]. The strikingly perinuclear localization of these organelles in mutant cells suggested that their trafficking was impaired (Fig. 5). This impairment could block the release of surfactant, as supported by the accumulation of endogenous SP-B and exogenous expressed SP-B and SP-C within LBs in mutant cells (Fig. 8). Interestingly, a recent study revealed that both BORCS5-KO and BORCS7-KO A549 cells accumulate LAMP1-positive organelles (however, the potential LB nature of these organelles and the effects of these KOs on surfactant release was not examined) near the nuclei [45]. Moreover, Borcs5 KO and Borcs7 KO are both neonatal lethal in mice [46, 47]. Interestingly, the Borcs5-KO mouse showed atelectasis of the lungs and died of respiratory failure within 1 h of birth [46]. We speculated these trafficking defects might also occur in vivo and influence pulmonary surfactant production in corresponding mutant mice, and leading to perinatal death phenotypes. However, it would be improper to conclude that BORCS5, BORCS7 and GCN5L1/BORCS1 regulate LB trafficking through BORC complex since several findings did not support this conclusion. First, KXD1/BORCS4-KO mice are viable [48]. Second, BLOS2/BORCS2-KO zebrafish did not show a surfactant defect phenotype in the swimbladder [13]. Finally, BORCS6-KO A549 cells did not accumulate LAMP1-positive organelles perinuclearly [45]. Hence, the trafficking regulation machinery of LB likely only involves some BORC members. The interesting findings here indicate that further exploration of the trafficking machinery employed by LBs might shed new light on neonatal lethality due to respiratory problems.

### Supplementary Information

**Additional file 1: Figure S1. **GCN5L1 expression in the mouse lung. A. GCN5L1/Bloc1s1 expression at different developmental stages. B. Distribution of GCN5L1/Bloc1s1 expression at E18.5.

**Additional file 2: Figure S2. **Establishing GCN5L1-KO clones in MLE-12 cells. A. Schematic diagram showing the gene structure of GCN5L1 and the positions of the Cas9 targets, start and stop codons. B. Analysis of the efficiency of Cas9 targets by T7EI assay. C. Analysis of the efficiency of Cas9 target by Sanger sequencing. D. Mutant information in different clones.

**Additional file 3: Figure S3. **Sequencing maps of gDNA PCR products from different GCN5L1 mutant clones.

**Additional file 4: Figure S4. **GCN5L1-KO examination and cell viability test. A. GCN5L1 mRNA levels after GCN5L1 disruption. B. GCN5L1 protein levels after GCN5L1 disruption. C. MTT assays of WT and GCN5L1-KO clones (M2 and M29). Cells were plated and the absorbance at 450 nm was analyzed at 6, 24, 48, and 72 h. ns, not significant; **P < 0.01; t-test.

**Additional file 5: Figure S5. **Components and abundances of lipids secreted from WT and GCN5L1-mutant cells. A. The levels of the main lipid species secreted from WT and GCN5L1-mutant cells. B. The levels of the main subspecies secreted from WT and GCN5L1-mutant cells.

**Additional file 6: Figure S6.** RNA-seq and enrichment analysis of differentially expressed genes. A. Heat map representing the DEGs after GCN5L1 KO. B and C. The most enriched terms in the GO analysis of the DEGs after GCN5L1 KO. D and F. The most enriched terms in the KEGG pathway analysis of the DEGs after GCN5L1 KO.

**Additional file 7: Figure S7.** Exploration of potential regulators upstream of Cebpα. A. Representative immunoblot images of Foxa2 and Nkx2-1 protein expression levels in WT and GCN5L1-KO MLE-12 cells. B. Protein–protein interaction network of Cebpα derived from STRING database. The results are expressed as the mean ± SD of three independent experiments; ns, not significant.

**Additional file 8: Figure S8. **Modulating activity of the ROS–ERK–Foxo1–Cebpα axis or reconstruction of the mitochondrial expression of GCN5L1 failed to rescue surfactant production in GCN5L1 cells. A. Secretion of phospholipid in WT cells, M2 cells, M2 cells treated with DPI (M2 + DPI) or SCH772984 (M2 + SCH772984), or M2 cells infected with lentivirus expressing exogenous Foxo1 (M2 + Foxo1). B. Secretion of SP-B and SP-C in WT cells, M2 cells, M2 cells treated with DPI (M2 + DPI) or SCH772984 (M2 + SCH772984), M2 cells infected with lentivirus expressing exogenous Foxo1 (M2 + Foxo1) cells. C. Secretion of phospholipid in WT cells, M2 cells, and M2 cells infected with lentivirus expressing exogenous Cebpα (M2 + Cebpα) cells. D. Secretion of SP-B and SP-C in WT cells, M2 cells, and M2 cells infected with lentivirus expressing exogenous Cebpα (M2 + Cebpα) cells. The results are expressed as the mean ± SD of three independent experiments; n.s., not significant; *P < 0.05, **P < 0.01; t-test.

**Additional file 9: Figure S9. **Quantitation of accumulated vesicles in GCN5L1 mutant cells. A. Quantitation of Lysotracker Red-positive vesicles in WT cells, M2 cells, M29 cells and M2 + GCN5L1 cells. B. Quantitation of BODIPY phosphatidylcholine-positive vesicles in WT cells and M2 cells. C. Quantitation of Lamp1-positive vesicles in WT cells and M2 cells. D. Quantitation of Lysotracker Red and ABCA3-EGFP double-positive vesicles in WT cells and M2 cells. E. Quantitation of BODIPY phosphatidylcholine and ABCA3-mCherry double-positive vesicles in WT cells and M2 cells. F. Quantitation of Lamp1 and ABCA3-EGFP double-positive vesicles in WT cells and M2 cells. ns, not significant; **P < 0.01; t-test.

**Additional file 10: Figure S10. **Autophagy activity analysis of WT and GCN5L1 mutant cells. A. WT and M2 cells were transfected with a GFP–LC3-expressing plasmid before Lysotracker staining. B. Immunoblot image of WT, M2 and M29 cells with LC3 antibody.

**Additional file 11: Figure S11. **Localization of GCN5L1 in LBs. A. The co-localization scatter plot of anti-Lamp1 and anti-GCN5L1–FLAG signals. Pearson correlation coefficient is 0.17; Manders’ overlap coefficient is 0.51. B. Cells were transfected with GCN5L1–FLAG-expressing plasmid and stained with anti-Lamp1 and anti-FLAG antibodies.

**Additional file 12:** Table S1. Downregulated surfactant-related genes after GCN5L1 KO (extracted from RNA-seq results).

**Additional file 13:** Table S2. Expression of surfactant-related TFs after GCN5L1 KO (extracted from RNA-seq results).

**Additional file 14: **Table S3. Primers used in this study.

## Data Availability

The datasets used and/or analyzed during the current study are available from the corresponding author on reasonable request.

## Abbreviations

PS: Pulmonary surfactant
AT2: Alveolar type II epithelial cells
LB: Lamellar body
MVB: Multivesicular body
SP: Surfactant protein
ABCA3: Adenosine triphosphate (ATP)-binding cassette transporter A3
GCN5L1: GCN5 (general control of amino acid synthesis 5)-like 1
BLOC-1: Biogenesis of lysosome-related organelles complex 1
BORC: BLOC-1-related complex
LRO: Lysosome-related organelles
HPS: Hermansky–Pudlak syndrome
WT: Wild-type
KO: Knockout

## References

[1] Wang Y, Tang Z, Huang H, Li J, Wang Z, Yu Y, Zhang C, Li J, Dai H, Wang F, Cai T, Tang N (2018). Pulmonary alveolar type I cell population consists of two distinct subtypes that differ in cell fate. Proc Natl Acad Sci USA.

[2] Whitsett JA, Wert SE, Weaver TE (2015). Diseases of pulmonary surfactant homeostasis. Annu Rev Pathol.

[3] Whitsett JA, Wert SE, Weaver TE (2010). Alveolar surfactant homeostasis and the pathogenesis of pulmonary disease. Annu Rev Med.

[4] Wu K, Scott I, Wang L, Thapa D, Sack MN (2021). The emerging roles of GCN5L1 in mitochondrial and vacuolar organelle biology. Biochim Biophys Acta Gene Regul Mech.

[5] Pu J, Schindler C, Jia R, Jarnik M, Backlund P, Bonifacino JS (2015). BORC, a multisubunit complex that regulates lysosome positioning. Dev Cell.

[6] Huizing M, Helip-Wooley A, Westbroek W, Gunay-Aygun M, Gahl WA (2008). Disorders of lysosome-related organelle biogenesis: clinical and molecular genetics. Annu Rev Genomics Hum Genet.

[7] Donato V, Bonora M, Simoneschi D, Sartini D, Kudo Y, Saraf A, Florens L, Washburn MP, Stadtfeld M, Pinton P, Pagano M (2017). The TDH-GCN5L1-Fbxo15-KBP axis limits mitochondrial biogenesis in mouse embryonic stem cells. Nat Cell Biol.

[8] Wang L, Zhu L, Wu K, Chen Y, Lee DY, Gucek M, Sack MN (2020). Mitochondrial general control of amino acid synthesis 5 like 1 regulates glutaminolysis, mammalian target of rapamycin complex 1 activity, and murine liver regeneration. Hepatology.

[9] Lv T, Hu Y, Ma Y, Zhen J, Xin W, Wan Q (2019). GCN5L1 controls renal lipotoxicity through regulating acetylation of fatty acid oxidation enzymes. J Physiol Biochem.

[10] Thapa D, Manning JR, Stoner MW, Zhang M, Xie B, Scott I (2020). Cardiomyocyte-specific deletion of GCN5L1 in mice restricts mitochondrial protein hyperacetylation in response to a high fat diet. Sci Rep.

[11] Manning JR, Thapa D, Zhang M, Stoner MW, Traba J, Corey C, Shiva S, Sack MN, Scott I (2019). Loss of GCN5L1 in cardiac cells disrupts glucose metabolism and promotes cell death via reduced Akt/mTORC2 signaling. Biochem J.

[12] Lv T, Lu Y, Liu Y, Feng H, Li C, Sheng W, Cui Z, Zhu S, Gu X, Yang Z, Wan Q (2021). General control of amino acid synthesis 5-like 1-mediated acetylation of manganese superoxide dismutase regulates oxidative stress in diabetic kidney disease. Oxid Med Cell Longev.

[13] Chen T, Song G, Yang H, Mao L, Cui Z, Huang K (2018). Development of the swimbladder surfactant system and biogenesis of lysosome-related organelles is regulated by BLOS1 in Zebrafish. Genetics.

[14] Wasmeier C, Hume AN, Bolasco G, Seabra MC (2008). Melanosomes at a glance. J Cell Sci.

[15] Wang P, Chintagari NR, Narayanaperumal J, Ayalew S, Hartson S, Liu L (2008). Proteomic analysis of lamellar bodies isolated from rat lungs. BMC Cell Biol.

[16] Ridsdale R, Na CL, Xu Y, Greis KD, Weaver T (2011). Comparative proteomic analysis of lung lamellar bodies and lysosome-related organelles. PLoS ONE.

[17] Besnard V, Xu Y, Whitsett JA (2007). Sterol response element binding protein and thyroid transcription factor-1 (Nkx2.1) regulate Abca3 gene expression. Am J Physiol Lung Cell Mol Physiol.

[18] Matsuzaki Y, Besnard V, Clark JC, Xu Y, Wert SE, Ikegami M, Whitsett JA (2008). STAT3 regulates ABCA3 expression and influences lamellar body formation in alveolar type II cells. Am J Respir Cell Mol Biol.

[19] Yan C, Naltner A, Martin M, Naltner M, Fangman JM, Gurel O (2002). Transcriptional stimulation of the surfactant protein B gene by STAT3 in respiratory epithelial cells. J Biol Chem.

[20] Attarian SJ, Leibel SL, Yang P, Alfano DN, Hackett BP, Cole FS, Hamvas A (2018). Mutations in the thyroid transcription factor gene NKX2-1 result in decreased expression of SFTPB and SFTPC. Pediatr Res.

[21] Xu Y, Saegusa C, Schehr A, Grant S, Whitsett JA, Ikegami M (2009). C/EBP{alpha} is required for pulmonary cytoprotection during hyperoxia. Am J Physiol Lung Cell Mol Physiol.

[22] Wang L, Scott I, Zhu L, Wu K, Han K, Chen Y, Gucek M, Sack MN (2017). GCN5L1 modulates cross-talk between mitochondria and cell signaling to regulate FoxO1 stability and gluconeogenesis. Nat Commun.

[23] Qiao L, Shao J (2006). SIRT1 regulates adiponectin gene expression through Foxo1-C/enhancer-binding protein alpha transcriptional complex. J Biol Chem.

[24] Rached MT, Kode A, Silva BC, Jung DY, Gray S, Ong H, Paik JH, DePinho RA, Kim JK, Karsenty G, Kousteni S (2010). FoxO1 expression in osteoblasts regulates glucose homeostasis through regulation of osteocalcin in mice. J Clin Invest.

[25] Manning JR, Thapa D, Zhang M, Stoner MW, Traba J, McTiernan CF, Corey C, Shiva S, Sack MN, Scott I (2019). Cardiac-specific deletion of GCN5L1 restricts recovery from ischemia-reperfusion injury. J Mol Cell Cardiol.

[26] Thapa D, Manning JR, Stoner MW, Zhang M, Xie B, Sack MN, Scott I. Cardiomyocyte-specific deletion of GCN5L1 in mice limits ex vivo cardiac functional decline in response to a high fat diet. bioRxiv. 2019; 805283.

[27] Manning JR, Thapa D, Zhang M, Stoner MW, Traba J, Corey C, Scott I (2019). Loss of GCN5L1 in cardiac cells disrupts glucose metabolism and promotes cell death via reduced Akt/mTORC2 signaling. Biochem J.

[28] Guardia CM, Farías GG, Jia R, Pu J, Bonifacino JS (2016). BORC functions upstream of kinesins 1 and 3 to coordinate regional movement of lysosomes along different microtubule tracks. Cell Rep.

[29] Jia R, Guardia CM, Pu J, Chen Y, Bonifacino JS (2017). BORC coordinates encounter and fusion of lysosomes with autophagosomes. Autophagy.

[30] Farías GG, Guardia CM, De Pace R, Britt DJ, Bonifacino JS (2017). BORC/kinesin-1 ensemble drives polarized transport of lysosomes into the axon. Proc Natl Acad Sci USA.

[31] Bowman SL, Bi-Karchin J, Le L, Marks MS (2019). The road to lysosome-related organelles: insights from Hermansky-Pudlak syndrome and other rare diseases. Traffic.

[32] Sorokina EM, Feinstein SI, Milovanova TN, Fisher AB (2009). Identification of the amino acid sequence that targets peroxiredoxin 6 to lysosome-like structures of lung epithelial cells. Am J Physiol Lung Cell Mol Physiol.

[33] Islam MN, Gusarova GA, Monma E, Das SR, Bhattacharya J (2014). F-actin scaffold stabilizes lamellar bodies during surfactant secretion. Am J Physiol Lung Cell Mol Physiol.

[34] Cheong N, Madesh M, Gonzales LW, Zhao M, Yu K, Ballard PL, Shuman H (2006). Functional and trafficking defects in ATP binding cassette A3 mutants associated with respiratory distress syndrome. J Biol Chem.

[35] Yu L, McPhee CK, Zheng L, Mardones GA, Rong Y, Peng J, Mi N, Zhao Y, Liu Z, Wan F, Hailey DW, Oorschot V, Klumperman J, Baehrecke EH, Lenardo MJ (2010). Termination of autophagy and reformation of lysosomes regulated by mTOR. Nature.

[36] Scott I, Webster BR, Chan CK, Okonkwo JU, Han K, Sack MN (2014). GCN5-like protein 1 (GCN5L1) controls mitochondrial content through coordinated regulation of mitochondrial biogenesis and mitophagy. J Biol Chem.

[37] Zhang A, He X, Zhang L, Yang L, Woodman P, Li W (2014). Biogenesis of lysosome-related organelles complex-1 subunit 1 (BLOS1) interacts with sorting nexin 2 and the endosomal sorting complex required for transport-I (ESCRT-I) component TSG101 to mediate the sorting of epidermal growth factor receptor into endosomal compartments. J Biol Chem.

[38] Wikenheiser KA, Vorbroker DK, Rice WR, Clark JC, Bachurski CJ, Oie HK, Whitsett JA (1993). Production of immortalized distal respiratory epithelial cell lines from surfactant protein C/simian virus 40 large tumor antigen transgenic mice. Proc Natl Acad Sci USA.

[39] Lunding LP, Krause D, Stichtenoth G, Stamme C, Lauterbach N, Hegermann J, Ochs M, Schuster B, Sedlacek R, Saftig P, Schwudke D, Wegmann M, Damme M (2021). LAMP3 deficiency affects surfactant homeostasis in mice. PLoS Genet.

[40] Robichaud NAS, Khatami MH, Saika-Voivod I, Booth V (2019). All-atom molecular dynamics simulations of dimeric lung surfactant protein b in lipid multilayers. Int J Mol Sci.

[41] Morishita H, Kanda Y, Kaizuka T, Chino H, Nakao K, Miki Y, Taketomi Y, Guan JL, Murakami M, Aiba A, Mizushima N (2020). Autophagy is required for maturation of surfactant-containing lamellar bodies in the lung and swim bladder. Cell Rep.

[42] Falcón-Pérez JM, Starcevic M, Gautam R, Dell'Angelica EC (2002). BLOC-1, a novel complex containing the pallidin and muted proteins involved in the biogenesis of melanosomes and platelet-dense granules. J Biol Chem.

[43] Zhou W, He Q, Zhang C, He X, Cui Z, Liu F, Li W (2016). BLOS2 negatively regulates Notch signaling during neural and hematopoietic stem and progenitor cell development. Elife.

[44] Tian JH, Wu ZX, Unzicker M, Lu L, Cai Q, Li C, Schirra C, Matti U, Stevens D, Deng C, Rettig J, Sheng ZH (2005). The role of Snapin in neurosecretion: snapin knock-out mice exhibit impaired calcium-dependent exocytosis of large dense-core vesicles in chromaffin cells. J Neurosci.

[45] Araki M, Ito K, Takatori S, Ito G, Tomita T (2021). BORCS6 is involved in the enlargement of lung lamellar bodies in Lrrk2 knockout mice. Hum Mol Genet.

[46] De Pace R, Britt DJ, Mercurio J, Foster AM, Djavaherian L, Hoffmann V, Abebe D, Bonifacino JS (2020). Synaptic vesicle precursors and lysosomes are transported by different mechanisms in the axon of mammalian neurons. Cell Rep.

[47] Snouwaert JN, Church RJ, Jania L, Nguyen M, Wheeler ML, Saintsing A, Mieczkowski P, Manuelde Villena FP, Armao D, Moy SS, Lorenzo DN, Koller BH (2018). A mutation in the Borcs7 subunit of the lysosome regulatory BORC complex results in motor deficits and dystrophic axonopathy in mice. Cell Rep.

[48] Yang Q, He X, Yang L, Zhou Z, Cullinane AR, Wei A, Zhang Z, Hao Z, Zhang A, He M, Feng Y, Gao X, Gahl WA, Huizing M, Li W (2012). The BLOS1-interacting protein KXD1 is involved in the biogenesis of lysosome-related organelles. Traffic.

[49] Garcia-Verdugo I, Ravasio A, de Paco EG, Synguelakis M, Ivanova N, Kanellopoulos J, Haller T (2008). Long-term exposure to LPS enhances the rate of stimulated exocytosis and surfactant secretion in alveolar type II cells and upregulates P2Y2 receptor expression. Am J Physiol Lung Cell Mol Physiol.

