# Supplementary figures and images for: GCN5L1 regulates pulmonary surfactant production by modulating lamellar body biogenesis and trafficking in mouse alveolar epithelial cells
Source: Cell Mol Biol Lett. 2023 Nov 7;28:90. doi: 10.1186/s11658-023-00506-0 (PMC10631113; doi:10.1186/s11658-023-00506-0)

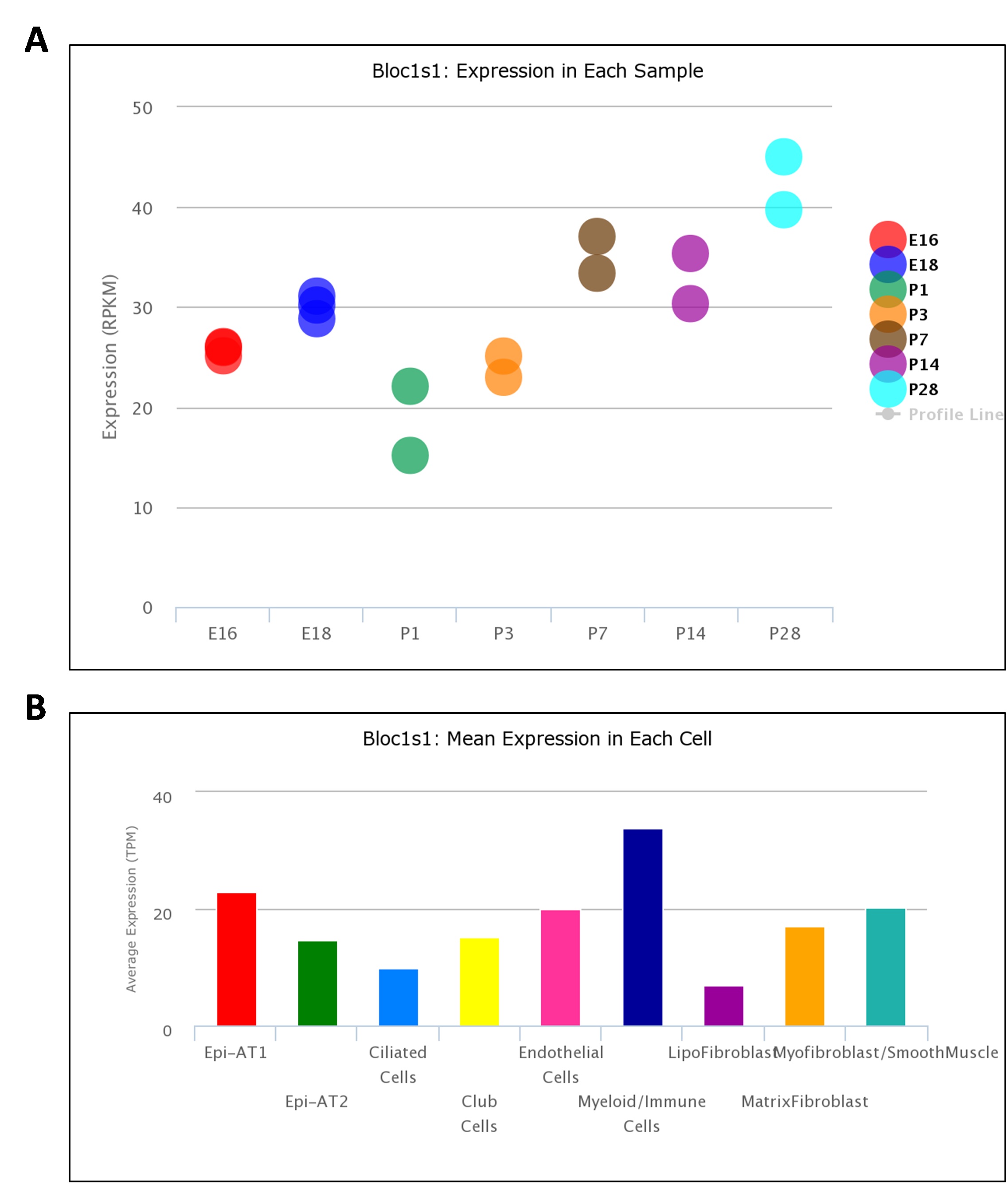

Supplement: Supplementary file 1 — Additional file 1: Figure S1. GCN5L1 expression in the mouse lung. A. GCN5L1/Bloc1s1 expression at different developmental stages. B. Distribution of GCN5L1/Bloc1s1 expression at E18.5. [file 11658_2023_506_MOESM1_ESM.jpg]

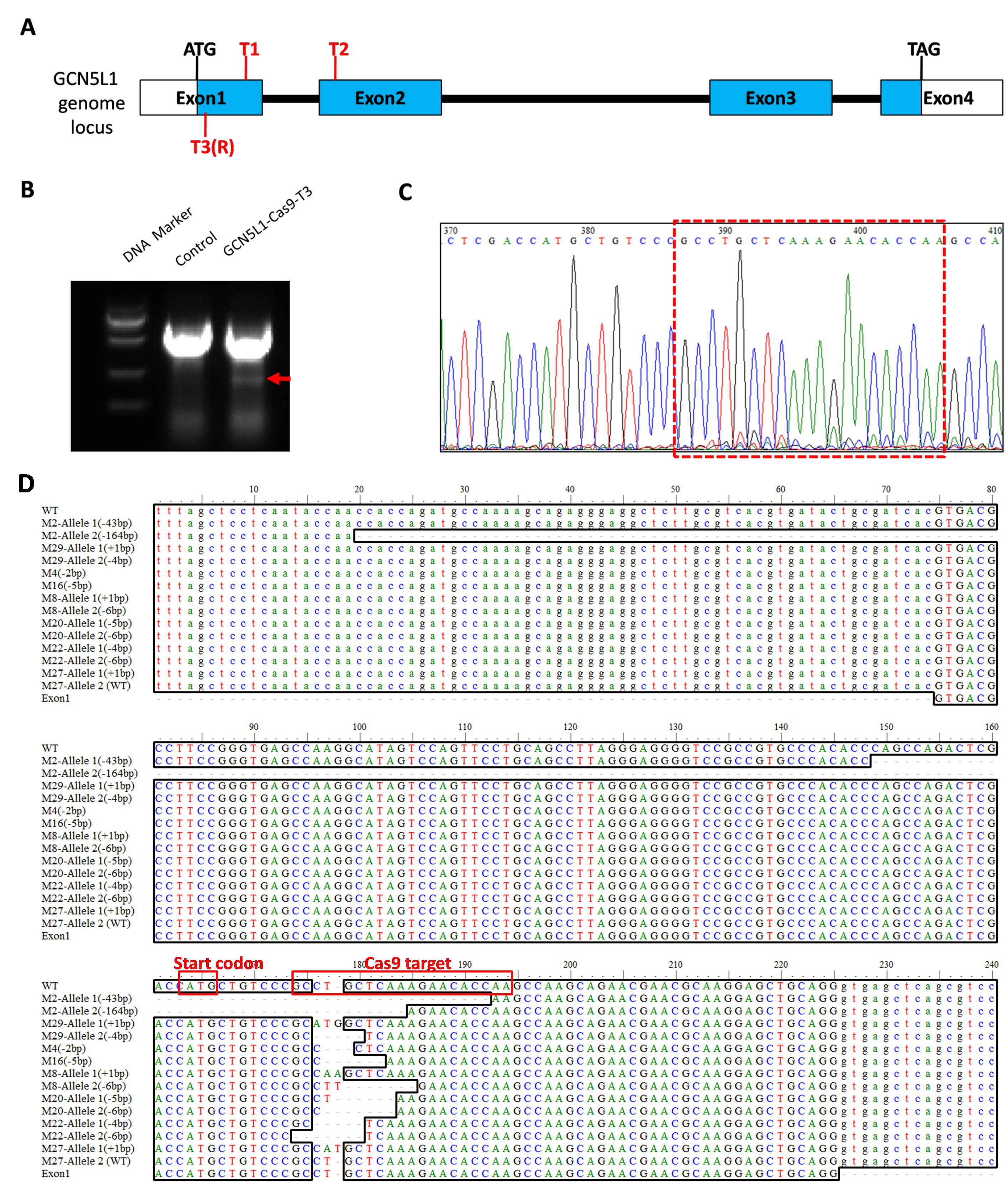

Supplement: Supplementary file 2 — Additional file 2: Figure S2. Establishing GCN5L1-KO clones in MLE-12 cells. A. Schematic diagram showing the gene structure of GCN5L1 and the positions of the Cas9 targets, start and stop codons. B. Analysis of the efficiency of Cas9 targets by T7EI assay. C. Analysis of the efficiency of Cas9 target by Sanger sequencing. D. Mutant information in different clones. [file 11658_2023_506_MOESM2_ESM.jpg]

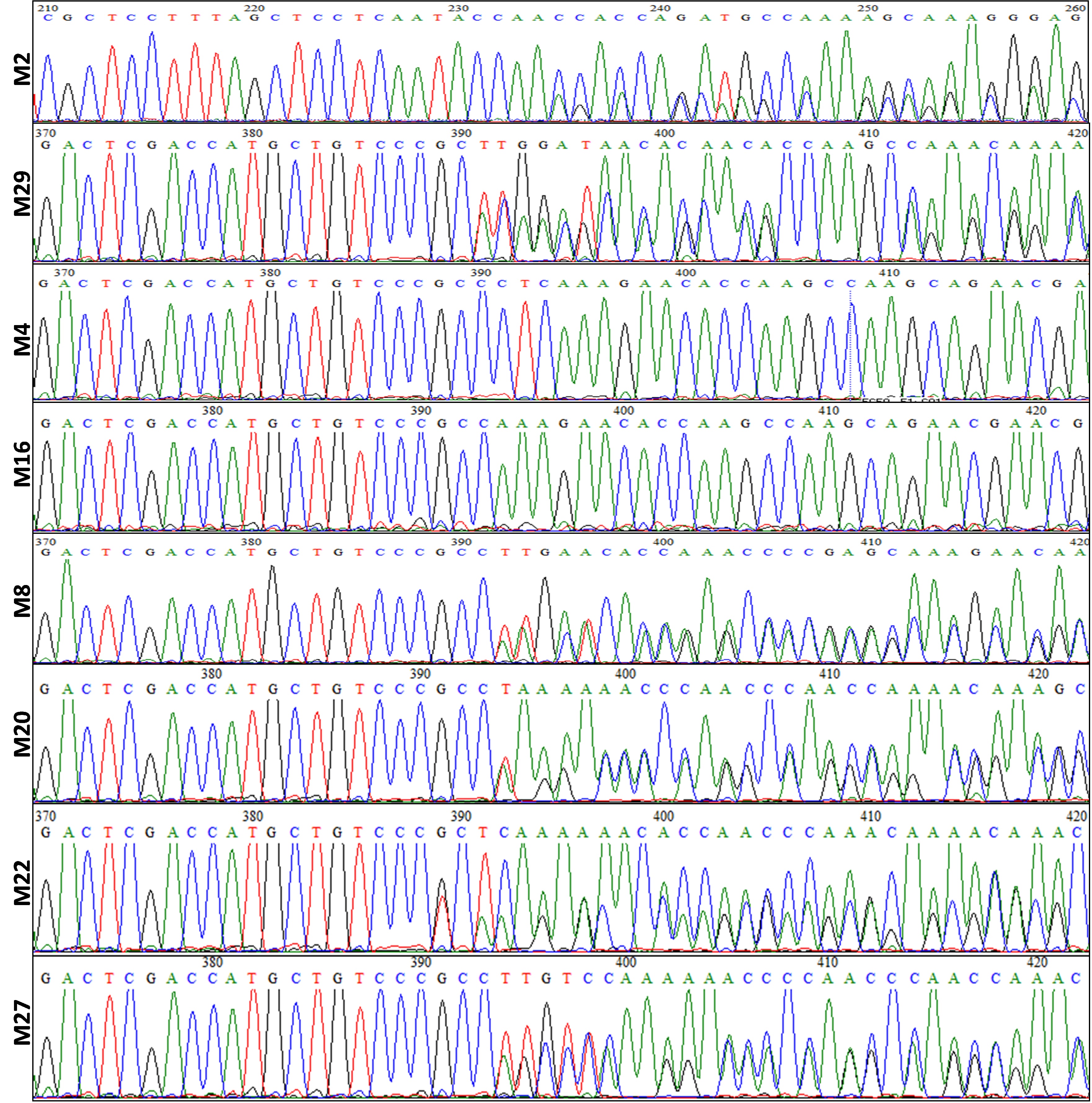

Supplement: Supplementary file 3 — Additional file 3: Figure S3. Sequencing maps of gDNA PCR products from different GCN5L1 mutant clones. [file 11658_2023_506_MOESM3_ESM.jpg]

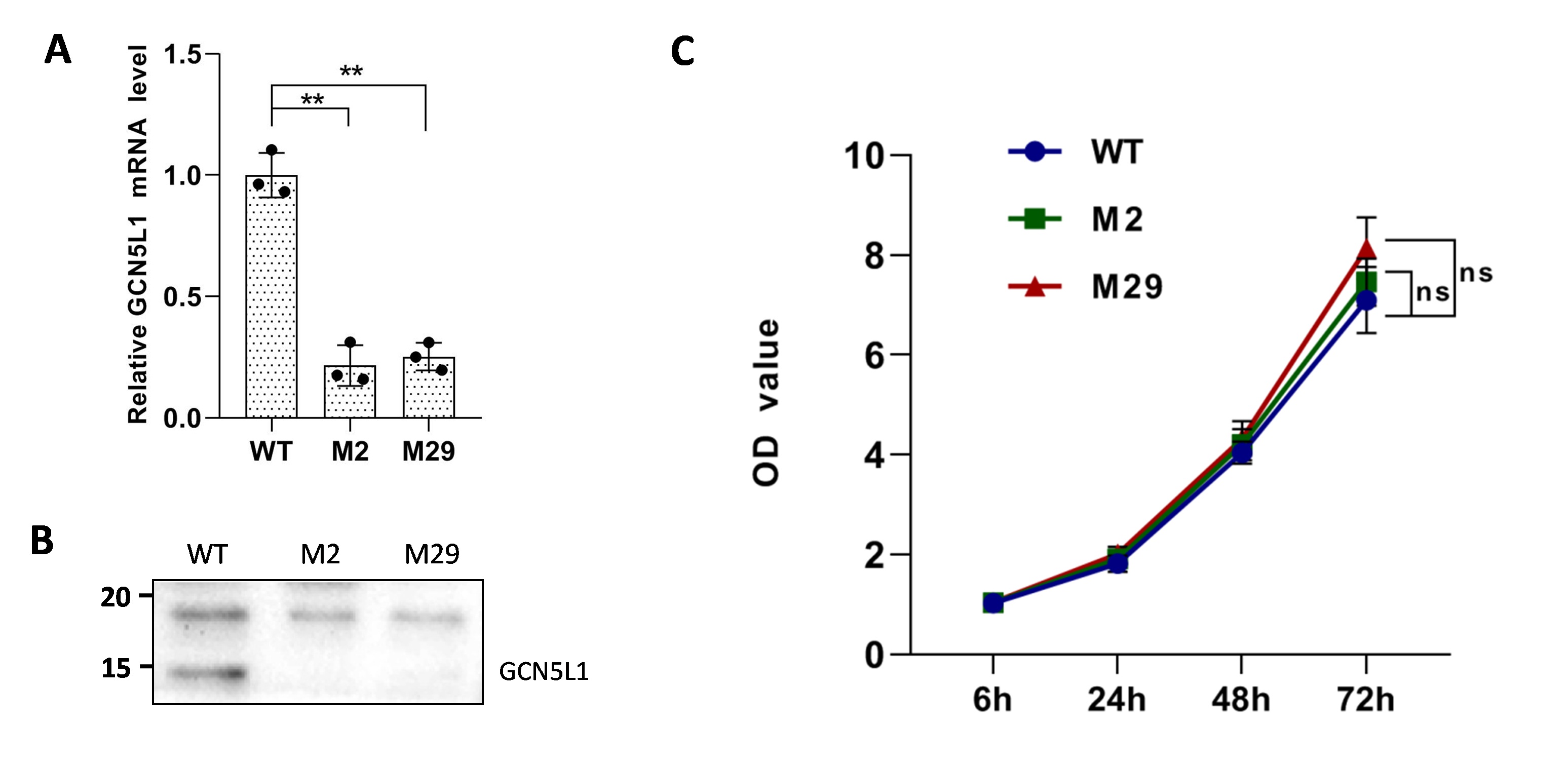

Supplement: Supplementary file 4 — Additional file 4: Figure S4. GCN5L1-KO examination and cell viability test. A. GCN5L1 mRNA levels after GCN5L1 disruption. B. GCN5L1 protein levels after GCN5L1 disruption. C. MTT assays of WT and GCN5L1-KO clones (M2 and M29). Cells were plated and the absorbance at 450 nm was analyzed at 6, 24, 48, and 72 h. ns, not significant; **P < 0.01; t-test. [file 11658_2023_506_MOESM4_ESM.jpg]

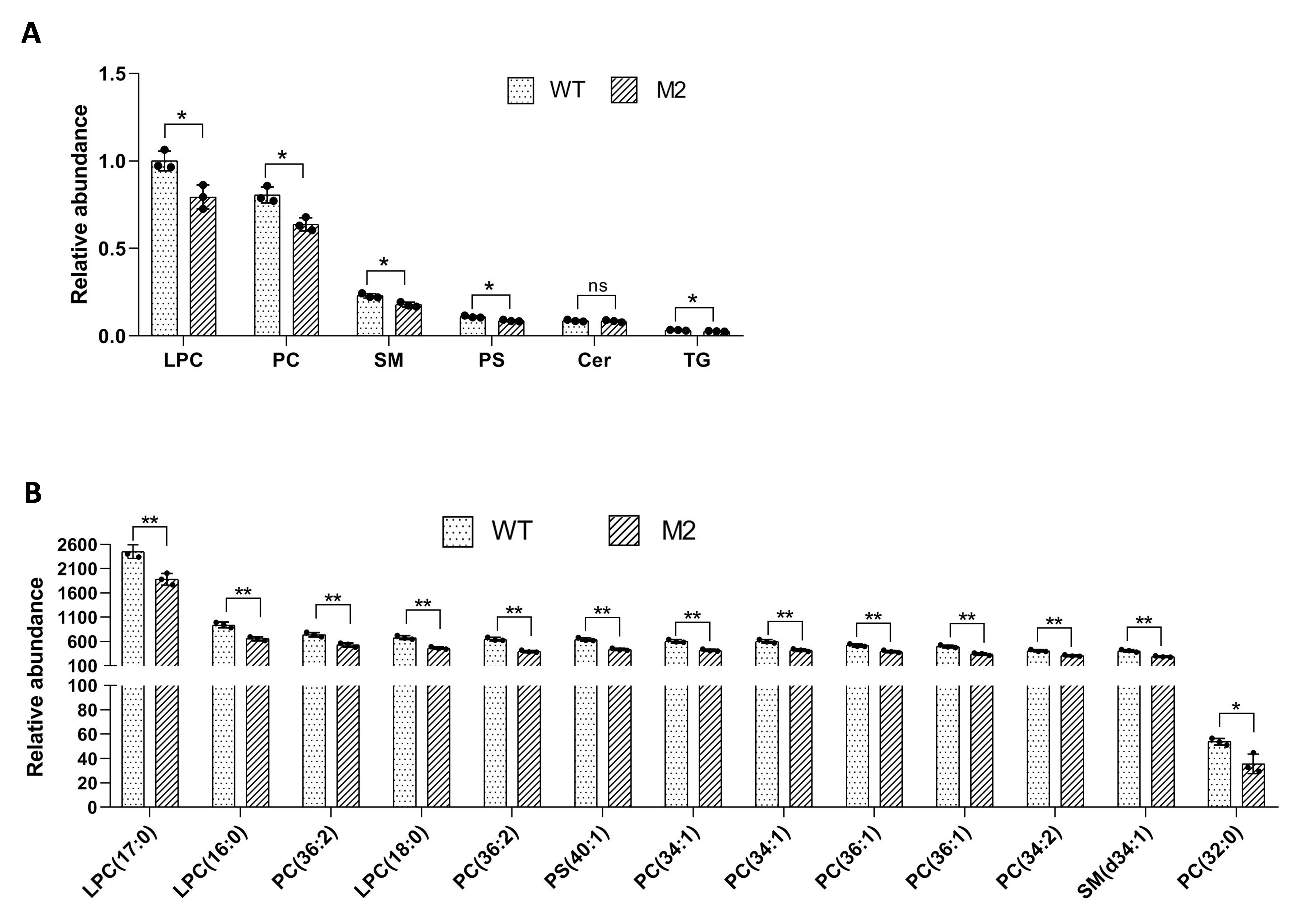

Supplement: Supplementary file 5 — Additional file 5: Figure S5. Components and abundances of lipids secreted from WT and GCN5L1-mutant cells. A. The levels of the main lipid species secreted from WT and GCN5L1-mutant cells. B. The levels of the main subspecies secreted from WT and GCN5L1-mutant cells. [file 11658_2023_506_MOESM5_ESM.jpg]

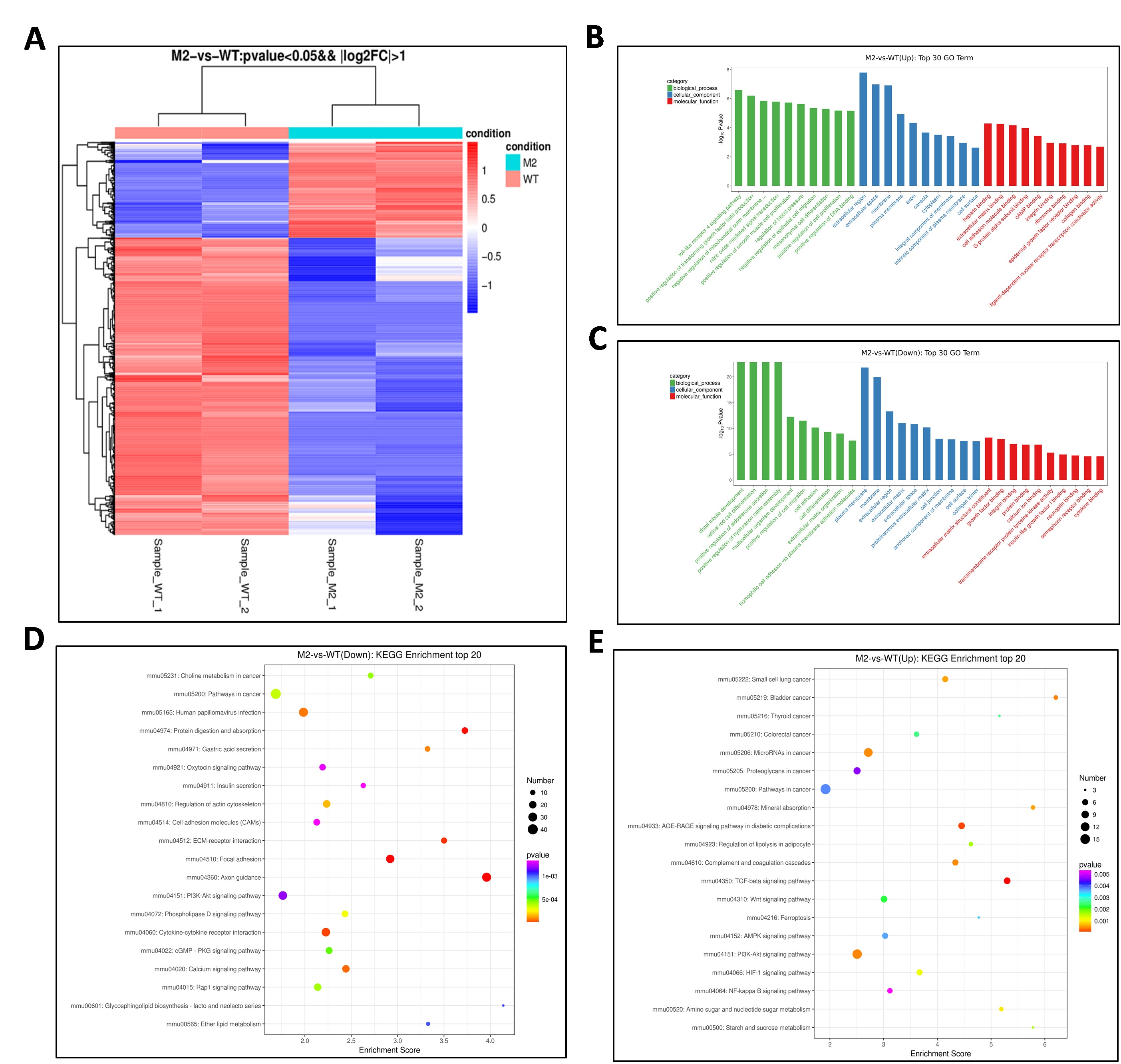

Supplement: Supplementary file 6 — Additional file 6: Figure S6. RNA-seq and enrichment analysis of differentially expressed genes. A. Heat map representing the DEGs after GCN5L1 KO. B and C. The most enriched terms in the GO analysis of the DEGs after GCN5L1 KO. D and F. The most enriched terms in the KEGG pathway analysis of the DEGs after GCN5L1 KO. [file 11658_2023_506_MOESM6_ESM.jpg]

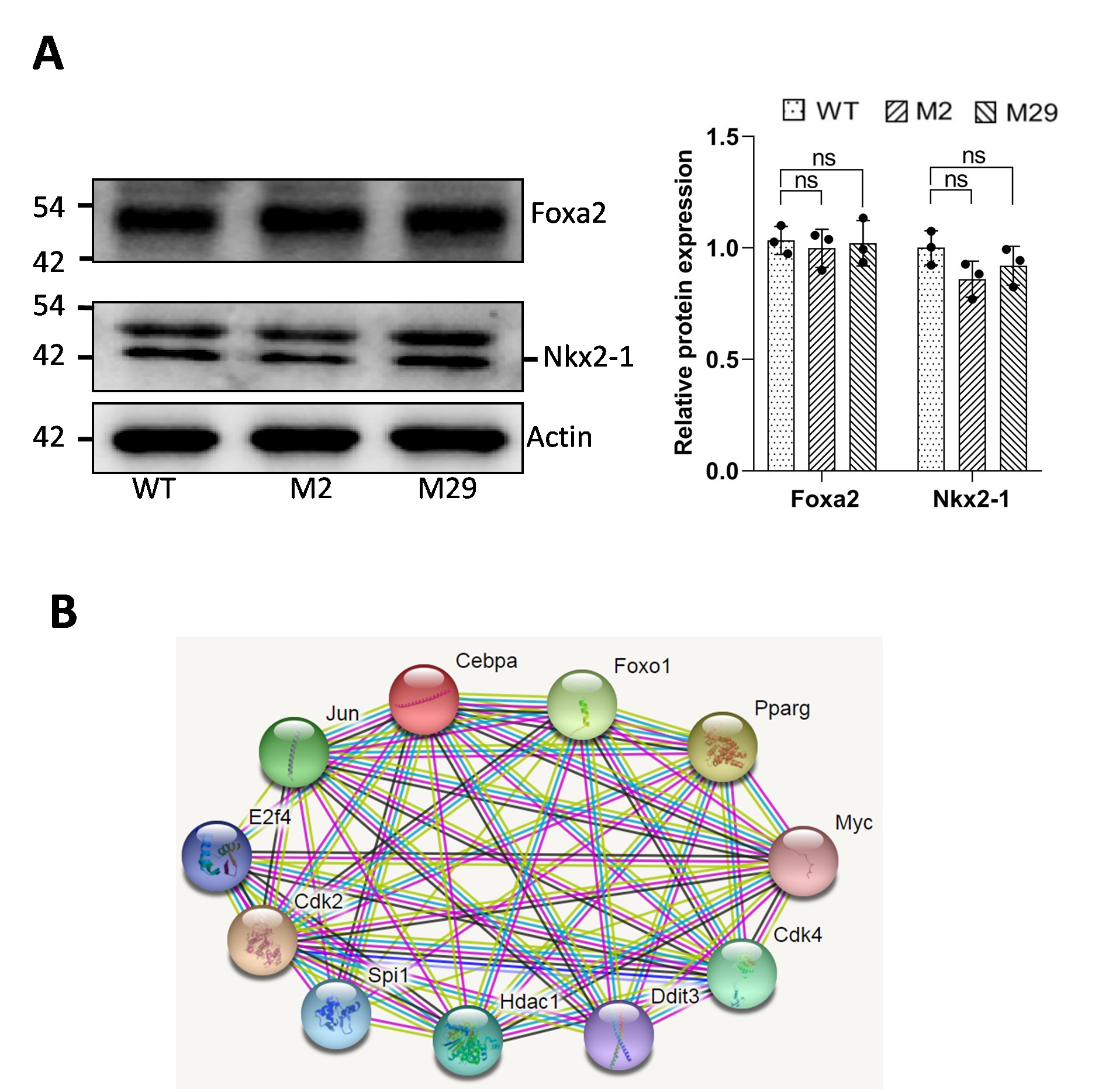

Supplement: Supplementary file 7 — Additional file 7: Figure S7. Exploration of potential regulators upstream of Cebpα. A. Representative immunoblot images of Foxa2 and Nkx2-1 protein expression levels in WT and GCN5L1-KO MLE-12 cells. B. Protein–protein interaction network of Cebpα derived from STRING database. The results are expressed as the mean ± SD of three independent experiments; ns, not significant. [file 11658_2023_506_MOESM7_ESM.jpg]

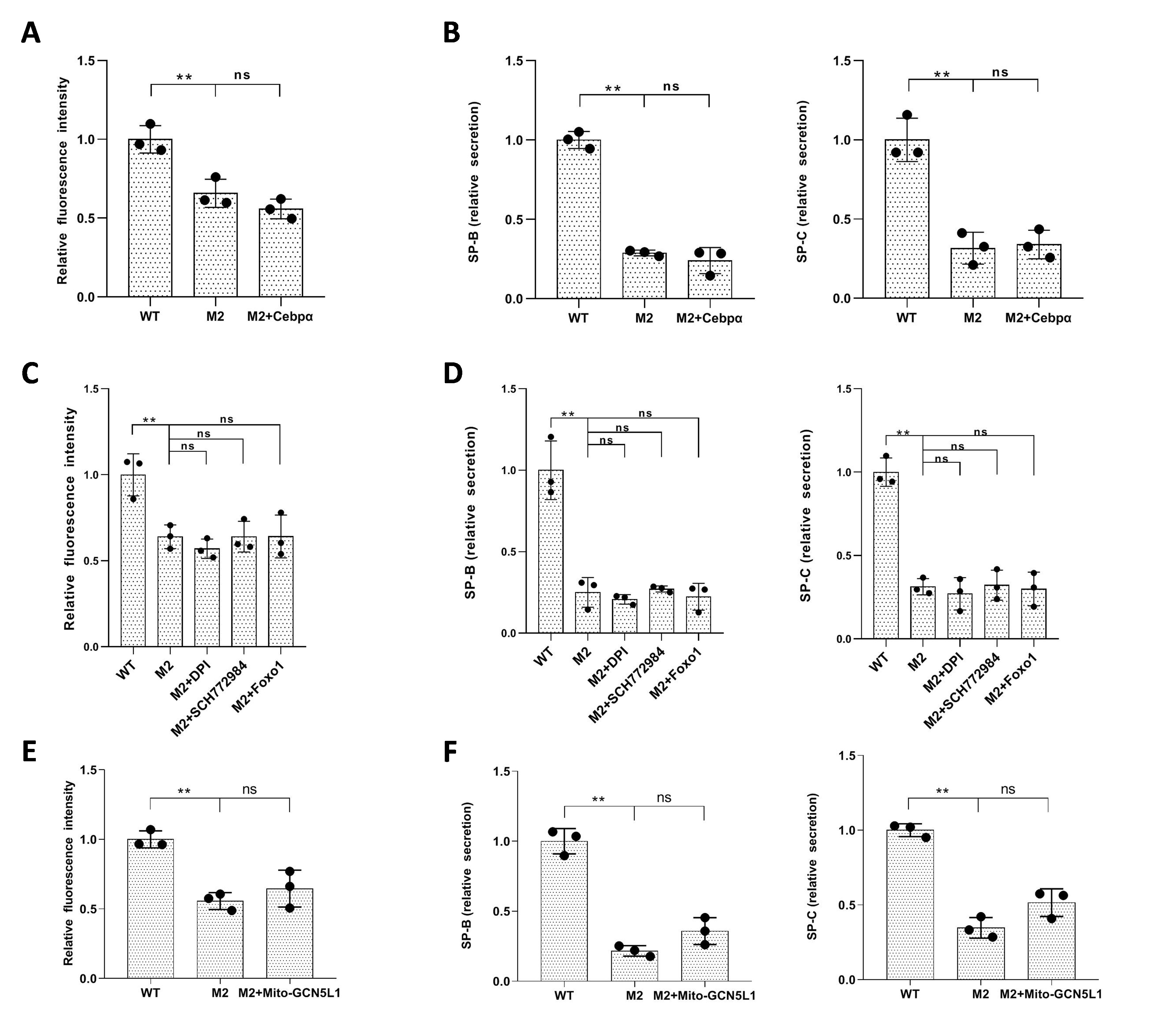

Supplement: Supplementary file 8 — Additional file 8: Figure S8. Modulating activity of the ROS–ERK–Foxo1–Cebpα axis or reconstruction of the mitochondrial expression of GCN5L1 failed to rescue surfactant production in GCN5L1 cells. A. Secretion of phospholipid in WT cells, M2 cells, M2 cells treated with DPI (M2 + DPI) or SCH772984 (M2 + SCH772984), or M2 cells infected with lentivirus expressing exogenous Foxo1 (M2 + Foxo1). B. Secretion of SP-B and SP-C in WT cells, M2 cells, M2 cells treated with DPI (M2 + DPI) or SCH772984 (M2 + SCH772984), M2 cells infected with lentivirus expressing exogenous Foxo1 (M2 + Foxo1) cells. C. Secretion of phospholipid in WT cells, M2 cells, and M2 cells infected with lentivirus expressing exogenous Cebpα (M2 + Cebpα) cells. D. Secretion of SP-B and SP-C in WT cells, M2 cells, and M2 cells infected with lentivirus expressing exogenous Cebpα (M2 + Cebpα) cells. The results are expressed as the mean ± SD of three independent experiments; n.s., not significant; *P < 0.05, **P < 0.01; t-test. [file 11658_2023_506_MOESM8_ESM.jpg]

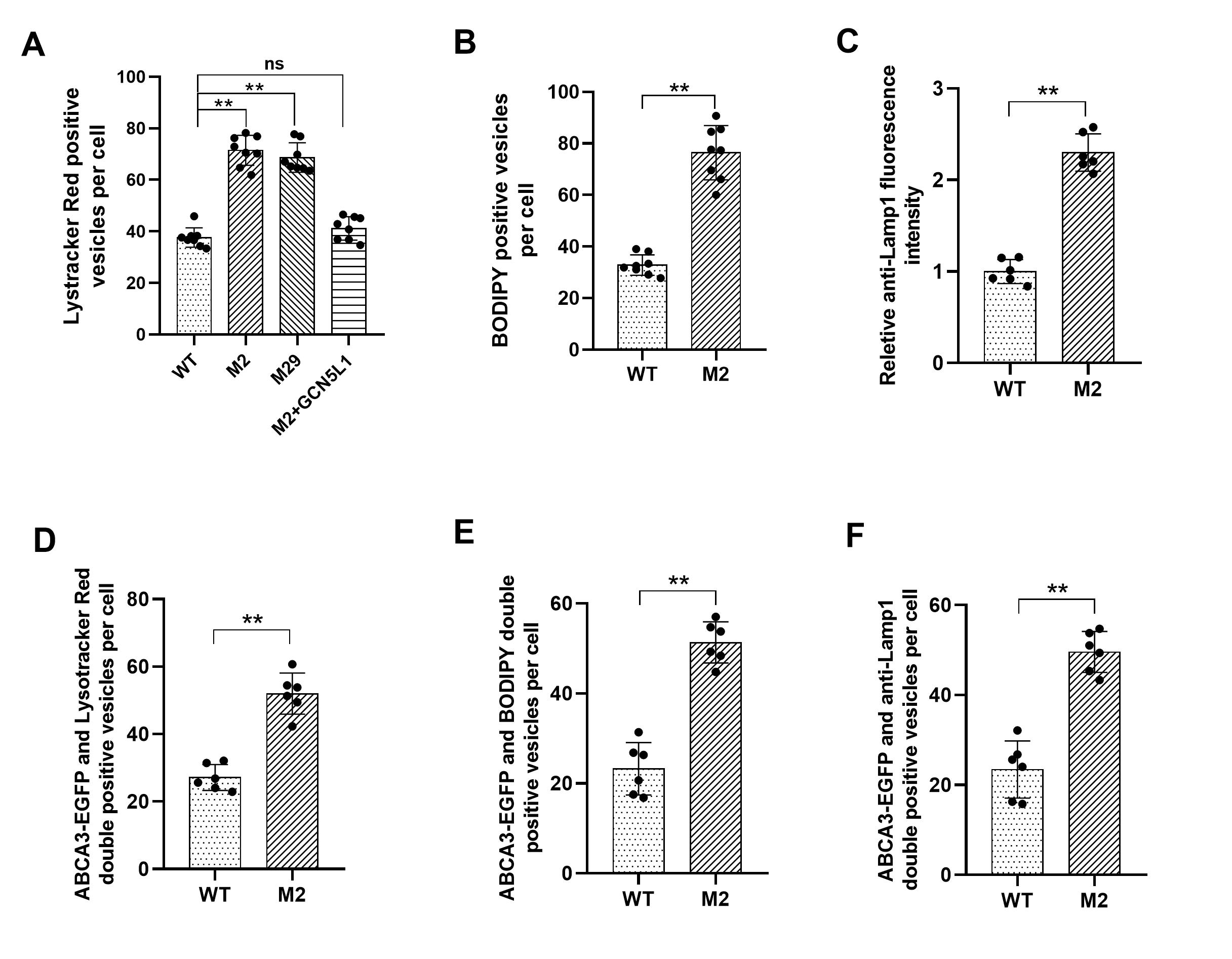

Supplement: Supplementary file 9 — Additional file 9: Figure S9. Quantitation of accumulated vesicles in GCN5L1 mutant cells. A. Quantitation of Lysotracker Red-positive vesicles in WT cells, M2 cells, M29 cells and M2 + GCN5L1 cells. B. Quantitation of BODIPY phosphatidylcholine-positive vesicles in WT cells and M2 cells. C. Quantitation of Lamp1-positive vesicles in WT cells and M2 cells. D. Quantitation of Lysotracker Red and ABCA3-EGFP double-positive vesicles in WT cells and M2 cells. E. Quantitation of BODIPY phosphatidylcholine and ABCA3-mCherry double-positive vesicles in WT cells and M2 cells. F. Quantitation of Lamp1 and ABCA3-EGFP double-positive vesicles in WT cells and M2 cells. ns, not significant; **P < 0.01; t-test. [file 11658_2023_506_MOESM9_ESM.jpg]

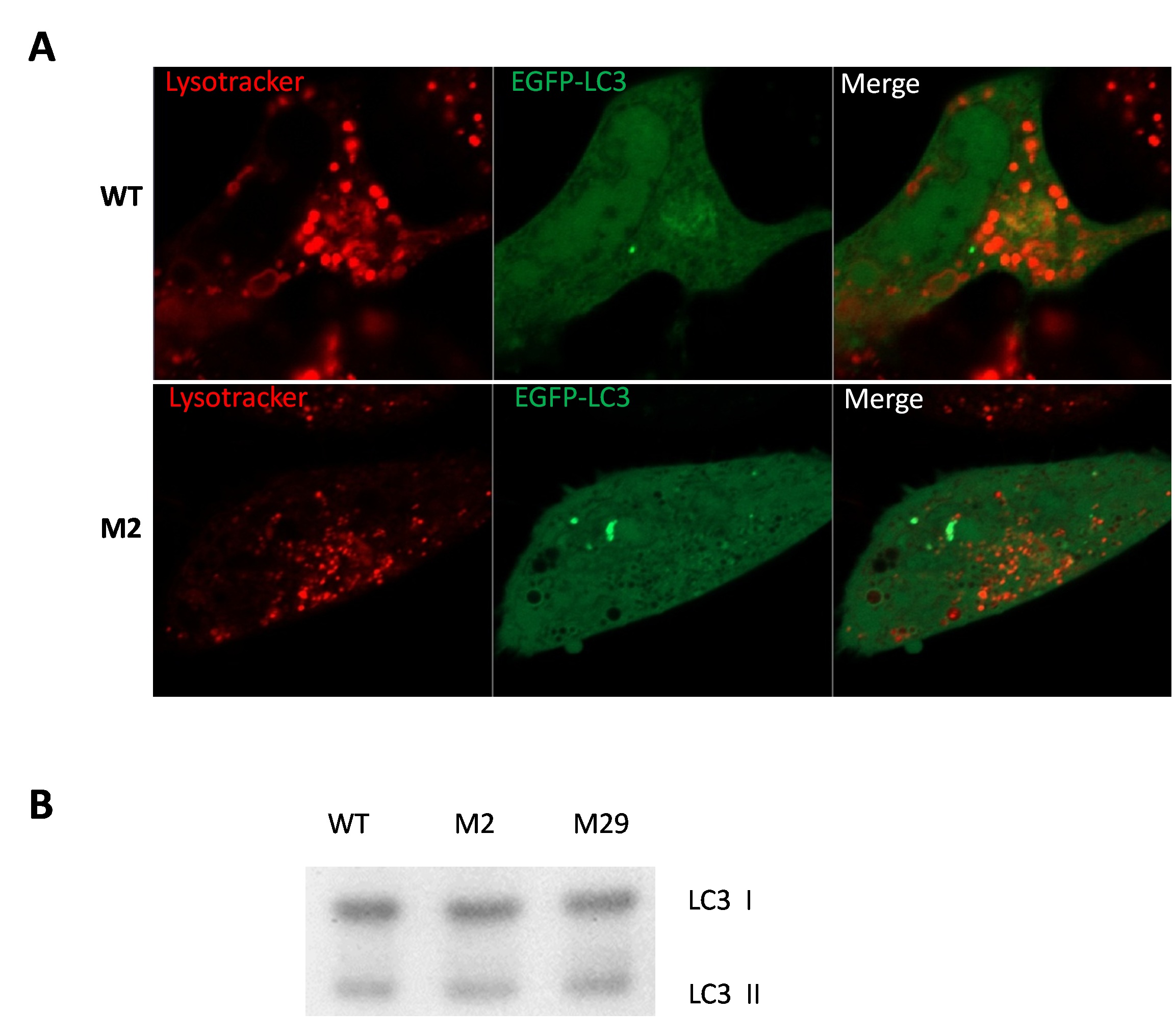

Supplement: Supplementary file 10 — Additional file 10: Figure S10. Autophagy activity analysis of WT and GCN5L1 mutant cells. A. WT and M2 cells were transfected with a GFP–LC3-expressing plasmid before Lysotracker staining. B. Immunoblot image of WT, M2 and M29 cells with LC3 antibody. [file 11658_2023_506_MOESM10_ESM.jpg]

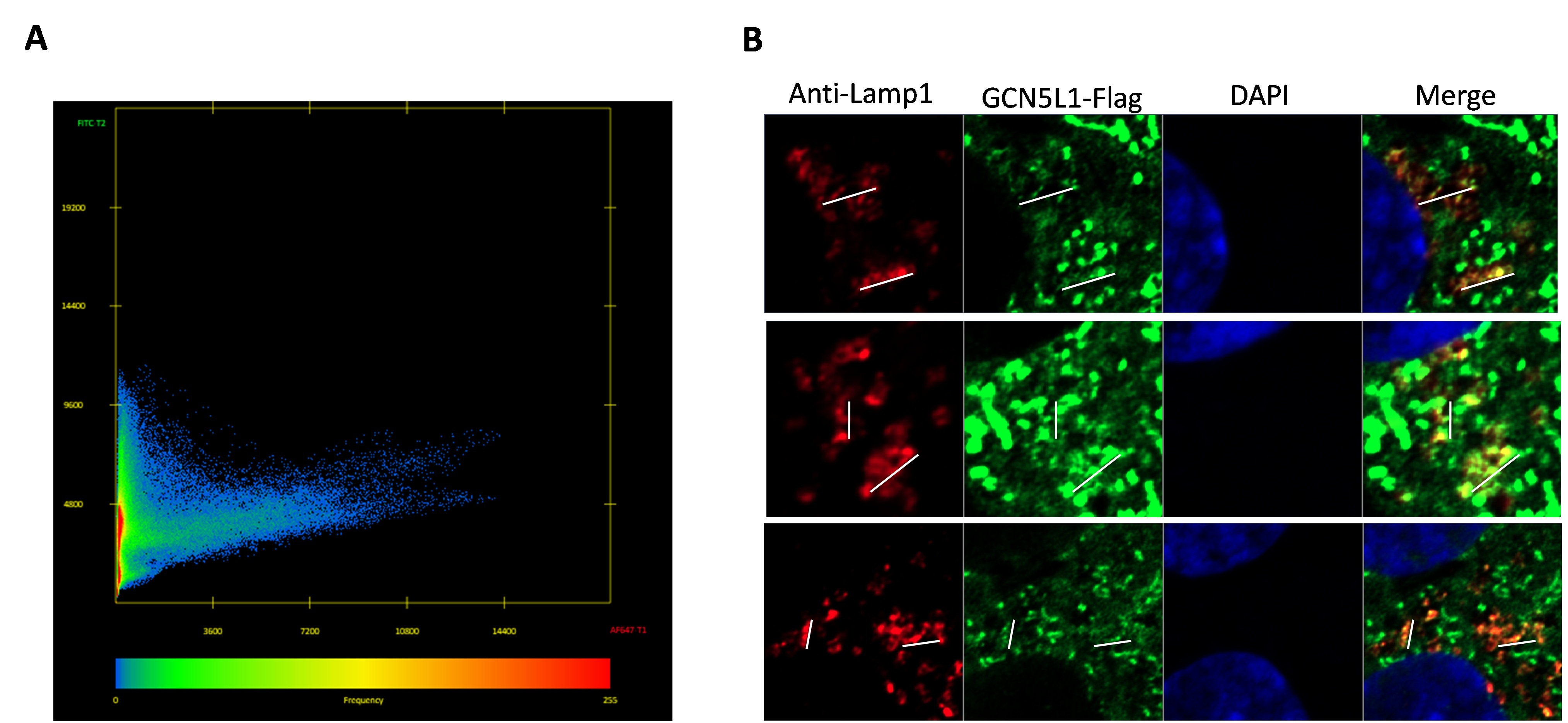

Supplement: Supplementary file 11 — Additional file 11: Figure S11. Localization of GCN5L1 in LBs. A. The co-localization scatter plot of anti-Lamp1 and anti-GCN5L1–FLAG signals. Pearson correlation coefficient is 0.17; Manders’ overlap coefficient is 0.51. B. Cells were transfected with GCN5L1–FLAG-expressing plasmid and stained with anti-Lamp1 and anti-FLAG antibodies. [file 11658_2023_506_MOESM11_ESM.jpg]
