# Supplementary material for: GCN5L1 regulates pulmonary surfactant production by modulating lamellar body biogenesis and trafficking in mouse alveolar epithelial cells
Source: Cell Mol Biol Lett. 2023 Nov 7;28:90. doi: 10.1186/s11658-023-00506-0 (PMC10631113; doi:10.1186/s11658-023-00506-0)
Supplement: Supplementary file 12 — Additional file 12: Table S1. Downregulated surfactant-related genes after GCN5L1 KO (extracted from RNA-seq results). [file 11658_2023_506_MOESM12_ESM.docx]

**Table S1 Downregulated surfactant-related genes after GCN5L1 KO (extracted from RNA-seq results)**

| Gene names | Expression in each sample (FPKM) | | | |
| --- | --- | --- | --- | --- |
|  | WT-1 | WT-2 | M2-1 | M2-2 |
| *Sftpb* | 5.02679 | 4.890607 | 0.849372 | 1.03142 |
| *Sftpc* | 23.4702 | 22.2113 | 6.7906 | 7.3144 |
| *Sftpa1* | 0 | 0 | 0 | 0 |
| *Sftpd* | 0 | 0 | 0 | 0 |
| *Abca3* | 17.4728 | 18.5116 | 10.4679 | 9.71531 |
| *Alpl* | 2.23594 | 1.70217 | 0.0447864 | 0.0228309 |
| *Susd2* | 15.7516 | 14.8166 | 2.55124 | 2.34624 |
| *C3* | 2.06184 | 2.21006 | 0.324135 | 0.40132 |
| *Aldoc* | 10.032 | 8.9959 | 3.54162 | 3.73949 |
| *Ptgfrn* | 2.88549 | 3.0339 | 0.989779 | 0.788469 |
| *Vnn1* | 5.20441 | 6.22035 | 1.35774 | 0.949559 |
| *Aldh3b1* | 7.51906 | 6.51731 | 3.58203 | 3.73968 |
| *Xpnpep* | 2.49009 | 2.26719 | 0.120661 | 0.0891133 |
| *Gsn* | 61.9416 | 55.7905 | 21.5541 | 19.3101 |
