# Supplementary material for: GCN5L1 regulates pulmonary surfactant production by modulating lamellar body biogenesis and trafficking in mouse alveolar epithelial cells
Source: Cell Mol Biol Lett. 2023 Nov 7;28:90. doi: 10.1186/s11658-023-00506-0 (PMC10631113; doi:10.1186/s11658-023-00506-0)
Supplement: Supplementary file 13 — Additional file 13: Table S2. Expression of surfactant-related TFs after GCN5L1 KO (extracted from RNA-seq results). [file 11658_2023_506_MOESM13_ESM.docx]

**Table S2 Expression of surfactant-related TFs after GCN5L1 KO (extracted from RNA-seq results)**

| Gene names | Expression in each sample  (FPKM) | | | |
| --- | --- | --- | --- | --- |
|  | WT-1 | WT-2 | M2-1 | M2-2 |
| *Cebpa* | 5.13115 | 5.40761 | 1.33487 | 1.22187 |
| *Nkx2-1* | 83.7746 | 79.3726 | 79.1394 | 73.5956 |
| *Foxa2* | 17.711 | 17.4719 | 17.0477 | 16.6578 |
| *Gata6* | 36.5974 | 38.9176 | 32.5381 | 35.2617 |
| *Nfatc3* | 39.2287 | 42.0318 | 30.5076 | 36.0249 |
| *Stat3* | 25.023 | 22.5176 | 21.9437 | 22.5662 |
| *Srebf1* | 23.2872 | 20.1201 | 21.9625 | 17.9897 |
| *Srebf2* | 66.9399 | 60.2109 | 63.3301 | 65.3209 |
