# Supplementary material for: GCN5L1 regulates pulmonary surfactant production by modulating lamellar body biogenesis and trafficking in mouse alveolar epithelial cells
Source: Cell Mol Biol Lett. 2023 Nov 7;28:90. doi: 10.1186/s11658-023-00506-0 (PMC10631113; doi:10.1186/s11658-023-00506-0)
Supplement: Supplementary file 14 — Additional file 14: Table S3. Primers used in this study. [file 11658_2023_506_MOESM14_ESM.docx]

**Table S3 Primers used in this study**

| Primer names | Primer sequences |
| --- | --- |
| G-M-GCN5L1-exon1-F | CATTGCCAGGAGGTAAGGTG |
| G-M-GCN5L1-exon1-R | ACGGAGTTTCCAGAAGTGCC |
| Q-M-Actin-F | TGCTGTCCCTGTATGCCTCTG |
| Q-M-Actin-R | TGATGTCACGCACGATTTCC |
| Q-M-Cebpa-F | GCGCAAGAGCCGAGATAAAG |
| Q-M-Cebpa-R | CCTTGACCAAGGAGCTCTCAG |
| Q-M-Foxo1-F | GTGAAGAGCGTGCCCTACT |
| Q-M-Foxo1-R | TTGAGCATCCACCAAGAAC |
| Q-M-Sftpb-F | AGCGCTACACAGTTCTCCTGCTA |
| Q-M-Sftpb-R | GGGCCCATGGCATCCT |
| Q-M-Sftpc-F | ACCCTGTGTGGAGAGCTACCA |
| Q-M-Sftpc-R | TTTGCGGAGGGTCTTTCCT |
| Q-M-Abca3-F | ACAGCACCTCAAGAGCAAGTT |
| Q-M-Abca3-R | CTTTCTCCAGGATACCAAATACC |
| Q-M-Alpl-F | ATAACGAGATGCCACCAGAGG |
| Q-M-Alpl-R | TCAGTGCGGTTCCAGACATAG |
| Q-M-Susd2-F | TGTGAGGGTGACCGTTTC |
| Q-M-Susd2-R | TTCCTTATGCCCGTTTGC |
| Q-M-C3-F | GTCCCTGTATGTCTCCGTCAC |
| Q-M-C3-R | AGCCTTTGCATTAGATCCCT |
| Q-M-Aldoc-F | CATCAACCGCTGCCCACTTC |
| Q-M-Aldoc-R | CCGCCATCTCCACTGCCTTC |
| Q-M-Ptgfrn-F | ACCCGCTCTTACCATCTACT |
| Q-M-Ptgfrn-R | GCCAGGTCACGCTTACAC |
| Q-M-Vnn1-F | GGGCTATGGGCATGAGGGT |
| Q-M-Vnn1-R | GCAGAATGGGATGGGTGGG |
| Q-M-Aldh3b1-F | GTGCCTGCCCTACAGAATG |
| Q-M-Aldh3b1-R | CCTGGAGTCGCTTGAAATG |
| Q-M-Xpnpep-F | ATCACCGCTGCTGTATGCC |
| Q-M-Xpnpep-R | GGTCCCGTGCTGGAAATCG |
| Q-M-Gsn-F | CCTAGTGGCTGATGAGAACC |
| Q-M-Gsn-R | CTGGGAGAACTGAAACCTG |
